# MISSA 2.0: an updated synthetic biology toolbox for assembly of orthogonal CRISPR/Cas systems

**DOI:** 10.1038/srep41993

**Published:** 2017-02-03

**Authors:** Hai-Yan Zhang, Xing-Hui Wang, Li Dong, Zhi-Ping Wang, Bing Liu, Jie Lv, Hui-Li Xing, Chun-Yan Han, Xue-Chen Wang, Qi-Jun Chen

**Affiliations:** 1 State Key Laboratory of Plant Physiology and Biochemistry, College of Biological Sciences, China Agricultural University, Beijing 100193, China

## Abstract

Efficient generation of plants carrying mutations in multiple genes remains a challenge. Using two or more orthogonal CRISPR/Cas systems can generate plants with multi-gene mutations, but assembly of these systems requires a robust, high-capacity toolkit. Here, we describe MISSA 2.0 (multiple-round *in vivo* site-specific assembly 2.0), an extensively updated toolkit for assembly of two or more CRISPR/Cas systems. We developed a novel suicide donor vector system based on plasmid RK2, which has much higher cloning capacity than the original, plasmid R6K-based system. We validated the utility of MISSA 2.0 by assembling multiple DNA fragments into the *E. coli* chromosome, and by creating transgenic *Arabidopsis thaliana* that constitutively or inducibly overexpress multiple genes. We then demonstrated that the higher cloning capacity of the RK2-derived MISSA 2.0 donor vectors facilitated the assembly of two orthogonal CRISPR/Cas systems including SpCas9 and SaCas9, and thus facilitated the creation of transgenic lines harboring these systems. We anticipate that MISSA 2.0 will enable substantial advancements in multiplex genome editing based on two or more orthogonal CRISPR/Cas9 systems, as well as in plant synthetic biology.

Plants bearing mutations in multiple genes of interest greatly facilitate analyzing epistatic relationships in genetic pathways and dissecting the roles of gene family members with redundant functions. Using CRISPR/Cas (clustered regularly interspaced short palindromic repeats/CRISPR-associated protein) system to induce multiplex mutations requires engineering multiple sgRNAs (single guide RNAs)^1^,^2^,^3^. Highly efficient expression of two or more sgRNAs has not been a problem, due to efficient methods for assembly of multiple sgRNAs^4^,^5^,^6^,^7^,^8^,^9^,^10^; therefore, it seems that, in an ideal situation, CRISPR/Cas9 can simultaneously edit an unlimited number of genomic sites. However, the current situation is far from ideal: efficient target search (and thus efficient editing) requires maintenance of the appropriate concentrations of each variant of the sgRNA-Cas9 complex in the nucleus^11^. The functional concentration of each variant of the sgRNA-Cas9 complex likely decreases in inverse proportion to the numbers of sgRNA variants or target sites, as the total concentration of all sgRNA-Cas9 complexes remains stable. Therefore, expression of multiple sgRNAs in a cell can affect the concentration of each variant of the sgRNA-Cas9 complex and thus reduce the efficiency of target search and relevant multiplex genome editing.

To solve the problem of reduced editing efficiency resulting from the co-existence of too many sgRNA variants in a cell, a few strategies could be employed. These strategies include increasing the expression level of Cas9, selecting sgRNAs that target two or more homologous genes^12^, crossing lines harboring mutations of a subset of the target genes, and re-transformation of mutants with a new CRISPR/Cas9 system (using mutant lines created by CRISPR/Cas9 but segregated from the transgenic components). Compared to these strategies based on one system, to have two or more orthogonal CRISPR/Cas systems work together would provide an alternative, possibly better strategy. However, efficient assembly of two or more orthogonal CRISPR/Cas systems into the same binary vector can hamper attempts to have orthogonal systems work together. To overcome this new obstacle, in this study, we report the development of MISSA 2.0 (for multiple-round *in vivo* site-specific assembly 2.0), second-generation MISSA technology, with improved, validated one-stop MISSA reagents for efficient assembly of two or more orthogonal CRISPR/Cas systems.

MISSA is a recyclable, *in vivo* site-specific DNA assembly method^13^. MISSA-standard for DNA assembly requires only two types of assembly interfaces (loxP-attL1-attL2 and loxP-attR2-attR1) for the assembly of large DNA fragments in any order and any direction. The standard has simple assembly rules, high assembly efficiency and flexibility, very low costs, and good compatibility with other standard methods, including BioBrick^14^, MoClo^15^, and GoldenBraid^16^. These advantages make MISSA very appropriate for the assembly of higher-order genetic units (HOGUs). In MISSA 2.0, we have extensively updated and improved the donor vectors, donor strains, recipient vectors, recipient strains, and related reagents. One of the key improvements is that we developed a novel suicide donor vector system based on plasmid RK2, which has higher cloning capacity (>300 kb)^17^,^18^. The new suicide donor vector system can better meet the requirements for the assembly of HOGUs with high molecular weight DNA, such as CRISPR/Cas systems. We first validated this new set of tools by assembling multiple DNA fragments into the *E. coli* chromosome, and by creating transgenic *Arabidopsis thaliana* that constitutively or inducibly overexpress multiple genes. We then demonstrated that the higher cloning capacity of the RK2-based MISSA 2.0 donor vectors significantly facilitated the assembly of two orthogonal CRISPR/Cas systems including SpCas9 and SaCas9, and thus facilitated the creation of transgenic lines harboring the two orthogonal CRISPR/Cas9 systems. We anticipate that MISSA 2.0 will contribute to the advancement in plant multiplex genome editing based on two or more orthogonal CRISPR/Cas9 systems, and may also enable advances in plant synthetic biology.

## Results

### MISSA 2.0 reagents: updated donor and recipient systems

**Figure 1:**
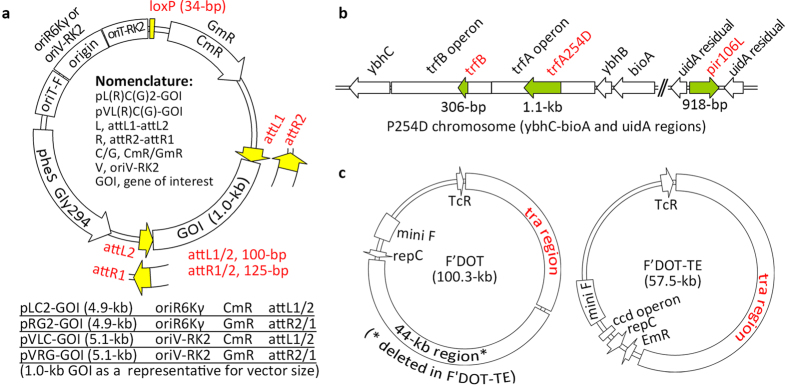
Physical maps and structures of the MISSA2.0 donor vectors and related constructs in the donor strains. **(a)** Physical maps and structures of two different sets of suicide donor vectors. The differential key features of the four types of donor vectors are indicated. *GmR*, gentamycin resistance gene; *CmR*, chloramphenicol resistance gene; oriT-F and oriT-RK2, *cis*-elements for conjugal transfer from plasmids F and RK2, respectively; oriR6Kγ and oriV, replication origins from plasmids R6K and RK2, respectively; *pheS Gly294*, a negative selectable marker that can be selected against with chlorophenylalanine (Cl-Phe). **(b)** Physical map and structure of the modified genomic regions of the host strain for both sets of suicide donor vectors. The *trfA* and *trfB* regions from RK2 required for the replication and stabilization, respectively, of oriV-based suicide donor vectors were inserted between *bioA*-*ybhB* and *ybhC* in the *E. coli* genome. *trfA254D* is a copy-up *trfA* mutant. The copy-up mutant gene *pir106L*, encoding a replication protein required for the propagation of high-copy oriR6Kγ-based suicide donor vectors, was inserted between the two *Mlu*I sites of *uidA* in the *E. coli* genome. **(c)** Physical maps and structures of the two F plasmid derivatives providing conjugational transfer function in donor strains. The 44-kb region between the *repC* and tra region of F′DOT was replaced with the *EmR* gene, resulting in F′DOT-TE. *EmR*, erythromycin resistance gene; *TcR*, tetracycline resistance gene; tra region, region providing conjugal transfer function; mini F, minimal region for the replication of the F plasmid.

**Figure 2:**
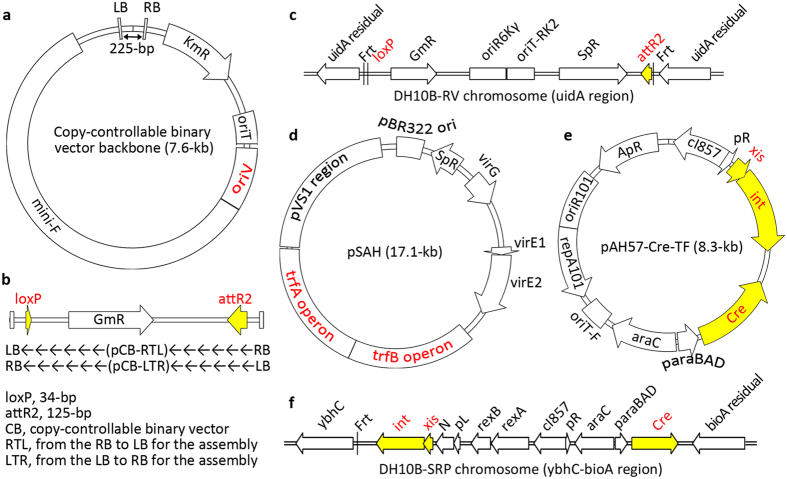
Physical maps and structures of recipient vectors and related constructs in recipient strains. **(a)** Physical maps of the backbone of copy-controllable recipient binary vectors. LB/RB, left/right border of T-DNA; See Fig. 1 for the oriT (oriT-RK2), oriV, KmR and mini-F. **(b)** Physical maps between RB and LB of pCB-RTL and pCB-LTR. **(c)** Physical map and structure of the modified genomic region of DH10B-RV, an *E. coli* genome-based recipient vector. The loxP-attR2 cassette flanked by two Frt sites was integrated into the *uidA* locus of the *E. coli* DH10B genome. *SpR*, spectinomycin resistance gene; see Fig. 1 for oriT-RK2, oriR6Kγ, and *GmR*. **(d)** Physical map and structure of pSAH, an *Agrobacterium* helper plasmid for propagation of the two binary vectors, pCB-RTL and pCB-LTR, in *Agrobacterium*. The pVS1 region, required for propagation of the plasmids in *Agrobacterium*; the *trfA* and *trfB* regions, required for the replication and stabilization, respectively, of the oriV-based binary vectors in *Agrobacterium. virG* and *virE1*/*2*, additional *Agrobacterium* virulence genes for enhancing infectivity of *Agrobacterium*. **(e)** Physical map and structure of conjugationally transferrable pAH57-Cre-TF, a helper plasmid providing two sets of site-specific recombination proteins. *ApR*, ampicillin resistance gene; repA101 and oriR101, the temperature-sensitive replication system of the plasmid; *cI857* and pR, required for heat shock-inducible expression of *xis* and *int* genes; *araC* and *paraBAD*, required for arabinose-inducible expression of *Cre*. **(f)** Physical map and structure of the modified genomic region of an engineered *E. coli* strain DH10B-SRD. The *xis* and *int* gene were provided by a modified defective phage lambda integrated into the attB locus (between *bioA*-*ybhB* and *ybhC*) of DH10B and the attL was replaced with Frt. *rexA*/*B* and *N*, residual phage lambda genes; *cI857* and pL, required for heat shock-inducible expression of *xis* and *int* genes.

The MISSA 2.0 reagents include a donor system (Fig. 1) and a recipient system (Fig. 2). The donor system comprises suicide donor vectors and donor strains harboring a conjugal transfer system; the recipient system comprises recipient vectors and recipient strains harboring a site-specific recombination system.

We added the oriT element from the F plasmid to the new donor vectors, which are compatible with the F plasmid conjugal transfer system^19^. We also used *pheS Gly294* as a negative selectable marker^19^ in place of the *sacB* marker used in the original version of MISSA. We named the new R6K-based suicide donor vectors pLC2-GOI and pRG2-GOI (Fig. 1a). To increase the insert size of donor vectors for cloning large HOGUs, we generated new suicide donor vectors named pVLC-GOI and pVRG-GOI (Fig. 1a) based on plasmid RK2, which has a cloning capacity of more than 300 kb^18^.

We generated three host strains, ABO, 203L, and 254D, for the propagation of the RK2-based suicide donor vectors (Fig. S1). Together with the two existing host strains BW20767^20^ and EPI300 (Epicentre), this gives five strains available for the propagation of the RK2-based suicide donor vectors. Four of the five strains (except EPI300) harbor the 3.2-kb trfB region from RK2, which contributes to RK2-based plasmid stability. Replication of RK2 depends on the RK2-encoded replication protein TrfA. The BW20767 and ABO strains harbor the wild-type *trfA* gene, but 203L, 254D, and EPI300 harbor the *trfA203L, trfA254D*, and *trfA203L* copy-up mutants, respectively, which increase the copy number by 3- to 20-fold, as compared with the wild-type *trfA*^21^. In EPI300, the arabinose operon tightly controls the expression of *trfA203L*. Therefore, L-Ara must be added to the culture media to induce the expression of *trfA203L*, thus allowing propagation of the RK2-based suicide donor vectors in EPI300. Comparison of copy numbers of the same RK2-based suicide vector (pVLC-GUS) found that the copy numbers increased significantly in EPI300, increased slightly in 254D, and showed no significant increase in 203L, as compared with those in BW20767 or ABO (Fig. S1). We generated the host strain P254D for the propagation of both RK2-based and R6K-based suicide donor vectors (Fig. 1b). Replication of R6K depends on the R6K-encoded replication protein Pir. BW20767 harbors the wild-type *pir* gene, but P254D harbors the *pir106L* copy-up mutant. To generate new donor strains harboring the conjugal transfer system, we replaced the 44-kb region of the plasmid F′DOT^19^ with the erythromycin resistance gene *EmR* to produce F′DOT-TE. The F′DOT-TE plasmid can be easily transformed into *E. coli* cells, whereas transformation of F′DOT is very hard. We generated the donor strain P254D-TE harboring the conjugal transfer system by introducing F′DOT-TE into P254D (Table 1).

We generated new recipient vectors named pCB-RTL and pCB-LTR (Fig. 2a,b), which are copy-controllable^21^. We also generated an *E. coli* chromosome-based recipient vector, into which we inserted the loxP-attR2 cassette via Flp/Frt site-specific recombination (Fig. 2c). The oriV element from RK2 in pCB-RTL and pCB-LTR has two functions: switching from single-copy to high-copy vectors in EPI300 when induced by L-Ara and acting as a replication origin in *Agrobacterium* engineered with the *trfA* gene harbored by the helper plasmid pSAH (Fig. 2d).

MISSA recipient strains must contain site-specific recombination systems based on both Cre/loxP and phage lambda. The site-specific recombination protein providers (SRPs) can be helper plasmid pAH57-Cre-TF (Fig. 2e), or *E. coli* chromosome DH10B-SRP (Fig. 2f). The different combinations of SRPs and recipient vectors produce several different recipient strains (Table 2).

**Figure 3:**
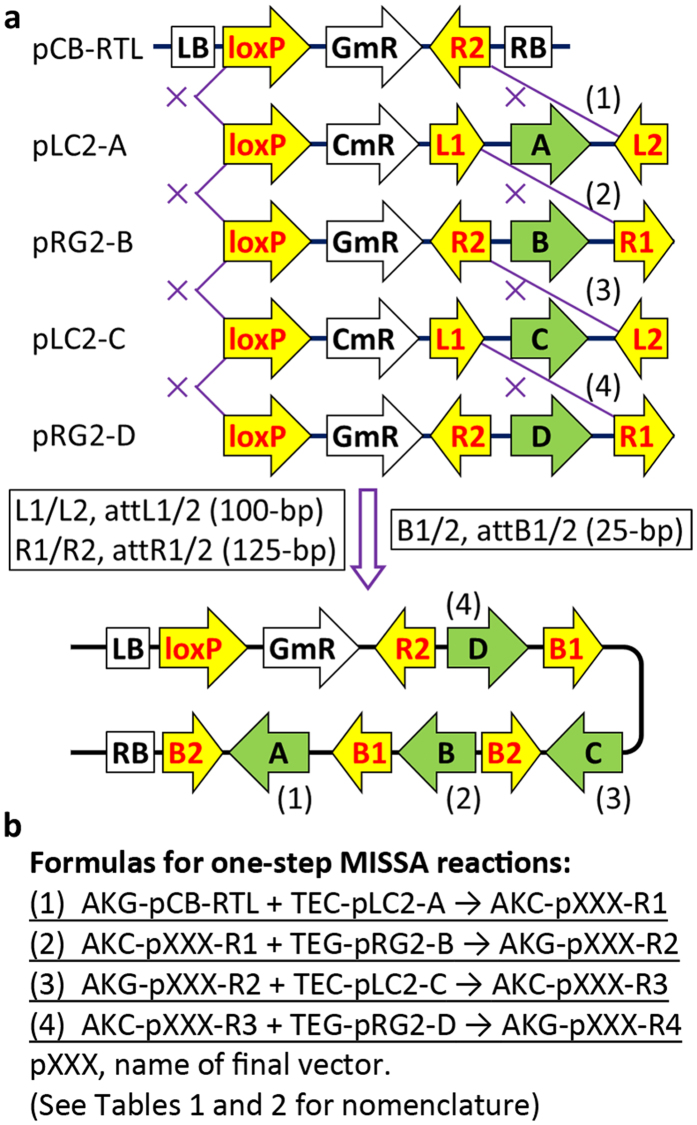
Schematic diagram of MISSA reactions. **(a)** Simplified schematic diagram displaying four rounds of MISSA reactions as an example. In each round of MISSA reaction, the loxP-attL2/attR1 fragment from a donor vector replaced the loxP-attR2/attL1 fragment in a recipient vector by site-specific recombination. The recipient vector can be an initial recipient vector (such as pCB-RTL), or any one of its derivatives resulting from MISSA reactions. Besides ABCD, the assembly orders for genes of interest (GOI) can be ADCB, CBAD, or CDAB. **(b)** Formulas for MISSA reactions. As an example, four rounds of MISSA reactions between recipient strains and donor strains are formulated. The AKG/AKC can be replaced with KG/KC or other combinations dependent on recipient strains used (Table 2). “+” represents mixing of recipient and donor strains, the arrows indicate the formation of new recipient strains. A/B/C/D, GOI-A/B/C/D. The letters before the first hyphen in a donor or a recipient stain name represent antibiotic resistances.

Simplifying DNA assembly rules as far as possible is a key part of standardization efforts for synthetic biology. MISSA has a simple, main rule for DNA assembly: site-specific recombination reactions must occur between two loxP sites, between attL1 and attR1 sites, or between attL2 and attR2 sites. The final constructs can be easily plotted once the assembly order is determined (Fig. 3). Removal of the antibiotic resistance genes from the final recipient vectors can be achieved by two-step MISSA reactions in the final round of assembly (Fig. S2).

To validate the MISSA 2.0 reagents, we performed two experiments, resulting in the final binary vectors pABA61 (Fig. S3) and pTEST51 (Fig. S4). We demonstrated the two vectors were assembled correctly by analyzing transgenic Arabidopsis (Figs S3 and S4).

### MISSA 2.0 enabled the assembly of DNA fragments into the *E. coli* chromosome

**Figure 4:**
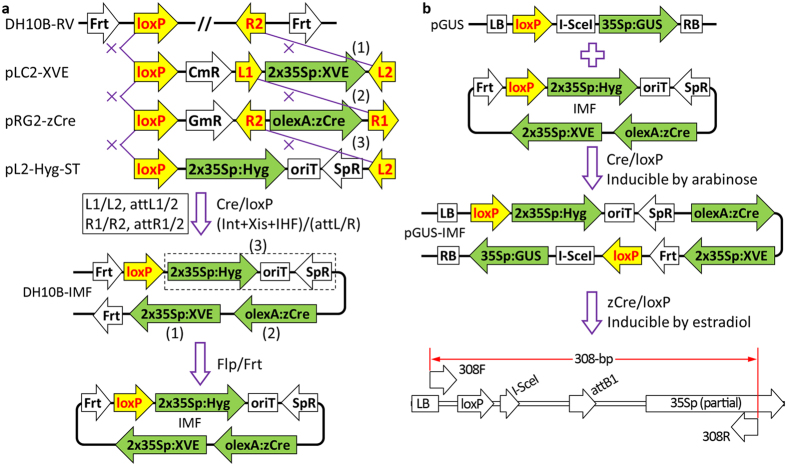
MISSA 2.0 allowed the assembly of DNA fragments into the *E. coli* chromosome. **(a)** Three rounds of MISSA reactions resulting in DH10B-IMF. The IMF (for inducible marker-free) construct was released from the chromosome in the circular form by Flp/Frt SSR reaction. **(b)** The circular IMF construct was transferred by the RK2 conjugative transfer system and re-integrated by Cre/loxP SSR reaction into another MISSA-assembled binary vector harboring genes of interest (*GUS* in this study as an example), resulting in the pGUS-IMF binary vector. The IMF construct can be released from the pGUS-IMF transgenic Arabidopsis cells when induced by estradiol, which can be detected by PCR with primers 308F/R. LB/RB, T-DNA left/right borders. oriT, conjugational transfer origin from RK2. *SpR*, spectinomycin-resistance gene.

One of the advantages of MISSA is that two or more DNA fragments can be efficiently assembled into the *E. coli* chromosome. To demonstrate this, we integrated a loxP-attR2 cassette into the *E. coli* chromosome, which thus became a huge MISSA recipient vector (Fig. 2, Table 2). We conducted three rounds of MISSA reactions and assembled three DNA fragments into the chromosome site (Fig. 4). The assembled construct was released from the chromosome in circular form via Flp/Frt-mediated SSR reaction (Fig. 4), and then the circular construct (named IMF, for inducible marker-free) was conjugally transferred to the MISSA recipient strain AK-pGUS, where IMF re-integrated into the binary vector pGUS via Cre/loxP-mediated site-specific recombination reaction (Fig. 4). We created transgenic Arabidopsis using the resultant construct, named pGUS-IMF. To validate pGUS-IMF, we introduced the plasmid into *Agrobacterium* strain COR308 via conjugal transfer, and then conducted Arabidopsis transformation. The plant selectable marker harbored by the IMF construct allowed screening of transgenic plants. IMF also harbors an XVE-inducible expression system of two transcriptional units (2x35 Sp:XVE and olexA-zCre). When induced by estradiol, the *zCre* gene, which is optimized with *Zea mays* codons and harbors an artificial intron, will be activated by XVE to express, and the IMF construct will be released from the Arabidopsis chromosome via zCre/loxP recombination (Fig. 4). This enables the generation of marker-free transgenic cells or plants. We laid leaves from six representative T1 transgenic Arabidopsis lines on an Murashige and Skoog (MS) agar plate supplemented with estradiol, and then we used PCR to detect marker-free cells from the leaves (Fig. 4). We obtained PCR fragments of the predicted size from the leaves of the six lines. We mixed the PCR fragments and sequenced them after purification. We aligned the sequences of the PCR fragments with the predicted sequence, which showed that the PCR fragment sequence agreed with the prediction (Fig. S5). These results demonstrated that the fragments of interest had correctly assembled into the *E. coli* chromosome.

### MISSA 2.0 enabled the creation of an inducible expression system for multiple genes in plants

Inducible expression systems offer numerous advantages and potential for a variety of applications^22^. For example, the use of an inducible system can allow the creation of transgenic plants harboring a transgene whose constitutive expression has serious effects on growth and development of the host plants. A reliable inducible expression system has been established based on XVE, a chimeric transcriptional activator^22^.

**Figure 5:**
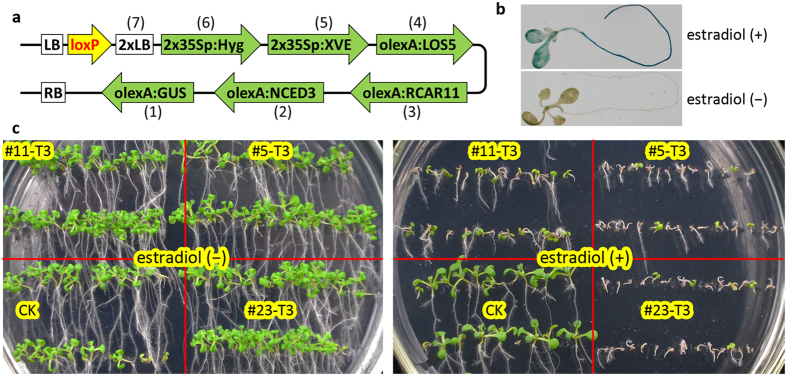
MISSA 2.0 facilitated the creation of an inducible expression system for multiple genes in plants. **(a)** Structure of the T-DNA region of piABA71 assembled by seven rounds of MISSA reactions. **(b)** GUS staining analysis of the inducible expression system. T3 seeds from a representative line were sown on MS agar plates containing 25 mg/L hygromycin, vernalized at 4 °C for three days, and grown under long-day conditions (16 h light/8 h dark) at 22 °C for seven days. The seedlings were then transferred to two MS agar plates (one supplemented with 10 μM α-estradiol) and allowed to grow for one day before GUS staining. Only two representative GUS-stained plants from the MS plate and the estradiol plate, respectively, are shown. **(c)** Germination and growth analysis of three representative lines under estradiol-induced (+) or non-induced (−) conditions. The seeds were sown on two MS agar plates supplemented with 25 mg/L hygromycin or 25 mg/L hygromycin plus 10 μM α-estradiol, vernalized at 4 °C for three days, and grown under long-day conditions (16 h light/8 h dark) at 22 °C for nine days before being photographed. CK, T3 plants derived from a hygromycin-resistant transgenic line.

MISSA 2.0 facilitated the creation of the inducible expression system for multiple genes. To demonstrate this, we generated donor vectors harboring specific transcriptional units in which the genes of interest or the *GUS* reporter gene were under the control of olexA (8xlexA operators fused with mini 35S promoter). We also generated two donor vectors harboring transcriptional units in which the *XVE* gene was driven by the 2x35S promoter. We conducted seven rounds of MISSA reactions and assembled the six transcriptional units: *XVE, NCED3, LOS5, RCAR11, GUS*, and *Hyg* (hygromycin resistance), and a fragment harboring two T-DNA left border (2xLB) sequences, into pCB-RTL, resulting in piABA71 (Fig. 5a). We used 2xLB to to enhance T-DNA cleavage at the LB sites, and thus to overcome possible integration of the vector backbone beyond LB into plant genome. To validate piABA71, we used conjugal transfer to introduce the plasmid into *Agrobacterium* strain GV3101 engineered with pSAH. We then created piABA71 transgenic Arabidopsis. We first tested the inducible expression system via GUS staining, which showed that the *GUS* gene was tightly controlled and responded efficiently to estradiol (Fig. 5b).

We then tested phenotypes when we induced strong expression of the genes of interest, which function in the abscisic acid (ABA) signaling pathway. Compared to control plants, germination and growth of the transgenic lines were seriously affected on the plates supplemented with estradiol, whereas they showed no difference in the absence of estradiol (Figs 5c and S6). We analyzed differentially expressed genes under induced and non-induced conditions by microarray analysis at the whole-genome scale. We treated the 7-day-old T3 seedlings derived from the #5 T1 line with or without estradiol for 3 days, and then submitted the total RNA samples for microarray analysis (Affymetrix ATH1). As expected, the expression levels of *NCED3*^23^, *LOS5*^24^, and *RCAR11*^25^,^26^ increased by 26-, 6-, and 4-fold, respectively, and the fold-changes increased with decreasing background expression levels of the three genes under non-induced conditions (Table S1). In addition, we expected that these genes would affect the expression of downstream genes in the ABA signaling pathway. Indeed, microarray data indicated that 248 genes were up-regulated (>2 fold, Table S1) whereas 305 genes were down-regulated (>2-fold, Table S2). Expression of two known ABA-inducible genes, *LTP4* and *LTP3*^27^, increased by 56- and 18-fold (Table S1), respectively, demonstrating that ABA levels and/or ABA sensitivity increased under induced conditions. To determine the main gene responsible for inhibition of seed germination, we assembled 3 constructs with combinations of two of the three genes (Fig. S7), and 3 constructs with the individual genes (Fig. S8). The results indicated that *NCED3*, rather than *LOS5* or *RCAR11*, is responsible for the inhibited seed germination. Together, these results demonstrated that MISSA 2.0 enables the creation of an inducible multiple-gene expression system in plants.

### MISSA 2.0 facilitated the assembly of orthogonal CRISPR/Cas9 systems

**Figure 6:**
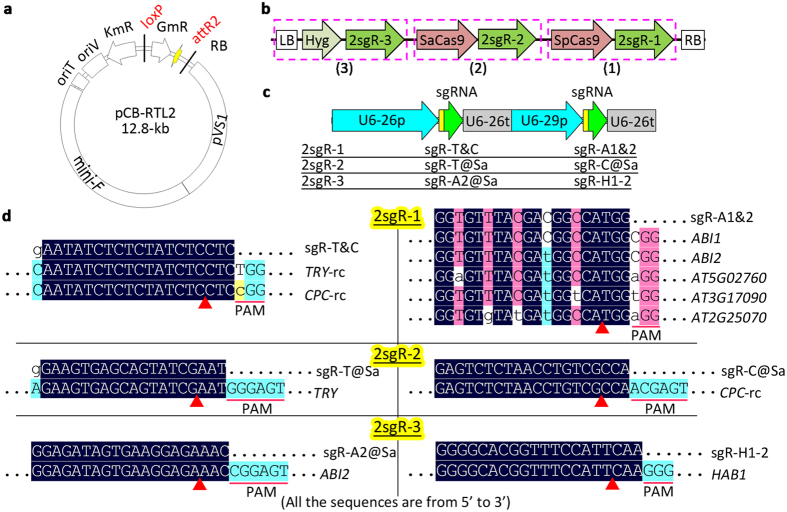
MISSA 2.0 facilitated the assembly of orthogonal CRISPR/Cas9 systems. **(a)** Physical map of a new recipient vector. See Fig. 2 for the mini-F, pVS1, oriT (oriT-RK2), oriV, KmR, GmR, and RB. **(b)** Structure of the T-DNA region of p2x3sgR assembled by three rounds of MISSA reactions. The SpCas9 and SaCas9 cassettes have the same fusion promoter (EC1.2en-EC1.1p) and terminator (pea *rbcS E9* terminator). sgR, sgRNA; 2sgR, two sgRNA cassettes; @Sa, SaCas9-specific sgRNAs. **(c)** Structure of the three 2-sgRNA-cassettes. **(d)** The alignment of the sgRNA with its target genes and potential off-targets. Only aligned regions of interest are displayed. -rc, reverse complement. The PAMs and the putative cleavage sites (red arrowheads) are indicated.

We next tested the MISSA 2.0 system for assembly of orthogonal CRISPR/Cas9 systems, using one system based on SpCas9 and one based on SaCas9. We were able to insert 6.3-kb fragment of the SpCas9 cassette into the donor vector pLC2-ccdB but failed in inserting the 8.1-kb fragment harboring the SpCas9 and sgRNA cassettes. Moreover, colonies harboring the ~11-kb pLC2-Cas9 plasmid grew much more slowly than normal ones. Therefore, we concluded that the R6K-derived donor vectors have a size limitation of ~11-kb. Thus, we used RK2-based donor vectors for assembly of two orthogonal CRISPR/Cas systems (Fig. 6). To simplify the CRISPR/Cas binary vectors, we generated a new recipient vector harboring the pVS1 region for propagation in *Agrobacterium* (Fig. 6). Another feature of pCB-RTL2 is that the vector backbone is free of the T-DNA LB, which overcomes the shortcoming of pCB-RTL: an unnecessary T-DNA region forms between the two LB sequences when the additional LB harbored in final donor vectors is assembled into the recipient vectors. The donor-harbored LB is added because it can keep unnecessary sequences (e.g. loxP-attL1/attR2 cassettes) out of the T-DNA region and thus block them from integrating into the genomic DNA in transgenic plants. We used the Golden Gate method to assemble two sgRNAs into each of the three intermediate donors to create final donors harboring sgRNAs targeting genes of interest. We then performed three rounds of MISSA reactions and assembled two orthogonal CRISPR systems into a binary vector (Fig. 6). We checked 6 colonies from every round of assembly and found that all 18 colonies were positive clones, demonstrating that MISSA 2.0 has high cloning efficiency.

To allow simple screening for mutants, we targeted the *TRY* and *CPC* genes, as double mutants in these genes have clustered leaf trichomes. We also targeted three of nine PP2C clade-A genes in ABA signaling pathway including *ABI1, ABI2*, and *HAB1* (Fig. 6). We first analyzed mutations of the target genes in T1 transgenic plants. Among 228 T1 lines, we obtained 17 likely *try cpc* double mutants (Fig. S9) and 2 mosaic mutants according to the phenotypes of clustered leaf trichomes. We then focused on the 17 lines and analyzed mutations in the target and potential off-target genes (Fig. 6). Cas9 from *Staphylococcus aureus* (SaCas9) is 1-kb smaller than the commonly used Cas9 from *Streptococcus pyogenes* (SpCas9), and has genome editing efficiencies comparable to those of SpCas9^28^. The SaCas9 use NNGRRT (R = G or A) as PAMs (protospacer-adjacent motifs) for target recognitions^28^. We analyzed SpCas9-induced mutations in *TRY, CPC*, and *HAB1*, and SaCas9-induced mutations in *TRY, CPC*, and *ABI2* by sequencing PCR fragments spanning the targets (Fig. 6) and identified mutation types based on sequencing chromatograms (Table S3). We also investigated sgR-A1&2/SpCas9-induced on-target mutations in *ABI1* and *ABI2* by *Nco*I digestion analysis, off-target mutations in *AT5G02760* and *AT2G25070* by *Nco*I digestion analysis, and off-target mutations in *AT3G17090* by direct sequencing PCR products (Fig. 6 and Table S3). We identified 8 likely homozygous or biallelic *abi1* mutants, and 1 heterozygous or chimeric off-target mutations in *AT5G02760* out of the 17 lines (Fig. S10), and found no mutations in the other 3 genes. Altogether, of six sgRNAs, all three SpCas9-associated sgRNAs produced high-frequencies of mutations whereas of the other three SaCas9-associated sgRNAs only 1 produced a medium frequency of mutations (Table S3).

We next analyzed mutations of two SaCas9 targets (*CPC* and *ABI2*) of 52 T2 lines derived from #4, and still failed to detect mutations. Although we used the CRISPscan tool^29^ to select potentially highly efficient sgRNAs except sgR-T&C, the tool, originally developed for SpCas9, might not produce efficient sgRNAs for the SaCas9 system. We further analyzed off-target mutations of the 52 T2 lines derived from #4. We observed a high frequency (84.6%, 44 out of 52 T2 plants) of off-target mutations in *AT5G02760* by *Nco*I digestion, whereas the mutation efficiency of the less-favorable target *ABI2* was 17.3% (9 out of 52 T2 plants) (Fig. S10). The high frequency of off-target mutations in *AT5G02760* of the T2 plants is likely due to germline transmission of the mutations from the T1 plant. In contrast, the homozygous or biallelic mutations in *ABI2* were newly generated in T2 one-cell stage embryo (#4–7). We observed no off-target mutations of *AT2G25070* and *AT3G17090* by *Nco*I digestion or direct sequencing of PCR products. We also analyzed off-target mutations of 41 T2 plants from #11, observing two off-target *AT5G02760* mutation lines (#11–13 and #11–21) and two less-favorable target *ABI2* mutation lines (#11–13 and #11–32) (Fig. S10). All together, our results demonstrated that MISSA 2.0 facilitated the assembly of two orthogonal CRISPR/Cas9 systems, although optimized sgRNAs will be required for efficient editing of SaCas9 targets.

## Discussion

Achieving a few proposed goals in plant synthetic biology, including engineering C4 or CAM photosynthesis^30^,^31^, or nitrogen fixation pathways^32^, will require the evolution of more efficient DNA assembly methods. DNA assembly occurs at different levels, from parts to TUs (transcriptional units), TUs to pathways, and pathways to networks and beyond; the quick and reliable assembly of standardized DNA parts into pathways and beyond can facilitate advances in synthetic biology^14^,^15^,^16^. MISSA is neither a seamless nor a multipartite assembly method; therefore, it is not appropriate for the assembly of parts into TUs. However, MISSA is very suitable for the assembly of higher-order genetic units (HOGUs), in that it leaves only minor, harmless scar sequences between HOGUs. Moreover, the high efficiency, flexibility, and standardization for the assembly of HOGUs by MISSA can offset the loss of efficiency caused by the fact that the assembly is not multipartite. Since the plasmid RK2, from which MISSA 2.0 suicide donor vectors (pVLC and pVRG) were derived, can hold DNA fragments >300 kb^17^,^18^, MISSA 2.0 can assemble large HOGUs, including TUs, composite TUs, or pathways and beyond. Indeed, the larger the HOGUs are, the more suitable MISSA 2.0 will be for their assembly.

Standardization of DNA parts, TUs, and beyond, together with simplification of assembly rules, can facilitate the advancement of synthetic biology. Plant synthetic biology generally requires functionally validated complete TUs, such as those for selectable markers and reporters, more frequently than it requires the corresponding parts. Since MISSA has only two standard assembly interfaces (loxP-attL1-attL2 and loxP-attR2-attR1) and simple assembly rules, MISSA donors can be used to produce standard TUs and other HOGUs. One advantage of the MISSA standards for TUs is their compatibility with different standards for parts, such as BioBrick^14^, MoClo^15^, and GoldenBraid^16^ through simple modification of multiple cloning sites of the MISSA 2.0 donors. Thus, MISSA 2.0 facilitates the advancement of synthetic biology by standardization of TUs and other HOGUs.

Although more and more orthogonal CRISPR/Cas systems can be used for plant genome editing^28^,^33^,^34^, efficient generation of multi-gene mutations in plants remains a challenge. To be efficient, first, the system should require one-time genetic transformation, with no additional genetic transformations. Second, multi-gene mutations should be derived from a single transgenic line (and its offspring) and require no crossing between different mutant lines. Third, to obtain homozygous mutants for multiple genes, the ratio of the number of mutant lines to total transgenic lines should be as high as possible and this should require only a few generations of transgenic lines. To achieve these, to have two or more orthogonal CRISPR/Cas systems work together might provide a better solution than using only one system. We demonstrated that using MISSA 2.0, we could assemble two orthogonal CRISPR/Cas9 systems easily and rapidly into a binary vector. Our results showed that three SpCas9-specific sgRNAs worked well and had a similar efficiency to that observed in our previous report^35^, suggesting that the system had not been affected by use of the SaCas9 system. We also demonstrated that SaCas9 worked well in plants. It is not unusual that only one out of three SaCas9-specific sgRNAs worked, since the mutation efficiency of different SpCas9 targets also varied over a wide range^29^. In fact, we had previously failed to detect mutations of some targets including *ABI1* (GAT TCC TTC ATG TTT AAA TT*A GG*), *ABI2* (TCA CTC TGA TTC ATC GAT CT*C GG*), and others using our egg cell promoter-controlled system^35^. Although we used the CRISPscan tool^29^ to select the three SaCas9-specific sgRNAs with relatively high scores (43 for *CPC*, 48 for *TRY*, and 64 for *ABI2*), the tool, originally developed for SpCas9, might not provide accurate scores for SaCas9. Another possibility is that activities of SaCas9 system were inhibited by SpCas9 system. However, it seems not to be the case since one SaCas9-specific sgRNA worked well. Furthermore, if SpCas9 and SaCas9 competed with each other for binding the SaCas9-specific sgRNAs, genome editing efficiencies of SpCas9 would also be affected. However, on the contrary, our results indicated that the SpCas9 system had not been affected. We are investigating performance of our maize codon-optimized SaCas9 by testing two targets in *ADH1*^36^ and other targets. We will perform further detailed investigations to reveal or exclude effects on each other of the two systems.

In addition to facilitating the assembly of two or more orthogonal CRISPR/Cas9 systems, MISSA 2.0 is also useful for assembly of a single CRISPR/Cas9 system harboring two or more sgRNAs located at different positions of the T-DNA. For example, we replaced the SaCas9 cassette with an mCherry cassette so that multiple sgRNAs could be separated by Cas9 and mCherry, and thus six or more sgRNAs could be efficiently assembled and separated (Supplementary File S1). The advantage of the spacing of multiple sgRNAs is that it can avoid homologous recombination-mediated loss of some sgRNAs due to too many repeated DNA elements placed together. Another advantage of the system is that the creation of sgRNA cassettes in donors is similar and simple, and thus the procedure for the generation of multiple sgRNAs is highly standardized. Our results indicated that off-target effects were exacerbated in T2 plants. The mCherry cassette would be useful for isolating Cas9-free Arabidopsis mutants^37^, and thus would help to avoid off-target mutations in the subsequent generations of mutants.

We also generated an SaCas9 cassette driven by germ-line-specific *SPL* promoter^38^; using this we could obtain EC1-SpCas9-based T1 or T2 homozygous mutants for some members of a gene family, and SPL-SaCas9-based T2 heterozygous mutants and T3 homozygous mutants for the other members (Supplementary File S1). In this way, we can investigate phenotypes of T3 plants, which harbor loss-of-function mutations of a whole gene family of interest. This strategy is useful and important, especially when mutations of a gene family are lethal or cause plant sterility. All together, our results show that MISSA 2.0 provides a platform for the assembly of two or more orthogonal CRISPR/Cas systems optimized in plants, and thus facilitates multiplex genome editing in plants.

## Methods

### Vector and *E. coli* strain construction

Detailed descriptions of the construction of vectors and *E. coli* strains^19^,^39^,^40^,^41^,^42^,^43^,^44^, and the formulas for the assembly of pABA61 or pTEST51 are provided in Supplementary Methods S1. The sequences of all primers used in this report are listed in Supplementary Table S4. Maps and sequences of vectors and *E. coli* chromosomal regions of interest are provided in Supplementary File S1.

### MISSA experiments

We performed each round of MISSA reactions using one or two steps. We used the two-step MISSA protocol for the final assembly to remove the donor vector-derived antibiotic resistance genes from the final recipient vectors; in other situations, we used the one-step MISSA protocol. We decided the types of antibiotics supplemented in culture media based on the names of the recipient strains in the MISSA reaction formulas.

One-step MISSA protocol: (1) Mix overnight cultures of donor and recipient strains (300 μl each), wash twice with LB, resuspend in 600 μl LB, heat shock at 42 °C for 1 h, and take 18 μl and 180 μl to spread onto two Cl-Phe agar plates (0.5% w/v yeast extract, 1% w/v NaCl, 0.4% w/v glycerol, 2% w/v agar and 10 mM D, L-p-Cl-Phe) supplemented with the appropriate antibiotics. Incubate at 32 °C for approximately 36 h. (2) Pick 18 well-isolated single colonies and streak on two plates, one supplemented with chloramphenicol (for AKG- or KG- recipient strains) or gentamycin (for AKC- or KC- recipient strains) for counter-selection. Incubate at 32 °C for approximately 12 h. (3) Identify assembled genes of interest for the counter-selection positive clones by colony PCR. Scrape off a little culture of the identified recipient strains, resuspend in liquid LB, and incubate the recipient strains at 32 °C and appropriate donor strains at 32 °C or 37 °C overnight for the next round of MISSA.

Two-step MISSA protocol: (1) Mix overnight cultures of donor and recipient strains (300 μl each), wash twice with LB, resuspend in 600 μl LB, incubate at 32 °C for 1 h, and take 18 μl and 180 μl to spread onto two LB agar plates, supplemented with the appropriate antibiotics (Fig. S2). Incubate at 32 °C for approximately 36 h. (2) Pick 18 well-isolated single colonies, put the cultures into 3 ml LB supplemented with 0.2% Glu, subject to heat shock at 42 °C for 1 h, and take 1.8 μl and 18 μl to spread onto two Cl-Phe agar plates supplemented with the appropriate antibiotics. Incubate at 32 °C for approximately 36 h. (3) Pick 18 well-isolated single colonies and streak on three plates, two supplemented with chloramphenicol or gentamycin, and spectinomycin, respectively, for counter-selection. Incubate at 32 °C for approximately 12 h. (4) Identify assembled genes of interest from the counter-selection positive clones by colony PCR.

### Assembly, release, transfer, and re-integration of the IMF construct

We performed three rounds of one-step MISSA reactions, producing the AS-IMF strain harboring the IMF construct in the *uidA* locus of the *E. coli* chromosome. The reactions can be formulated as follows: (1) AGS-RV + TEC-pLC2-XVE → AC-IMF-R1; (2) AC-IMF-R1 + TEG-pRG2-zCre → AG-IMF-R2; (3) AG-IMF-R2 + TES-pL2-Hyg-ST → AS-IMF. See Tables 1 and 2 for nomenclature. We screened out the temperature-sensitive SRP plasmid pAH57-Cre-TF by streaking AS-IMF cultures on a spectinomycin agar plate and growing cells at 42 °C to obtain single colonies (S-IMF) that had lost pAH57-Cre-TF. We performed two rounds of MISSA reactions, resulting in the creation of an AK-pGUS strain harboring the pGUS construct. The reactions can be formulated as follows: (1) AK-TAC-RTL + TEC-pLC2-GUS → AKC-pGUS-R1; (2) AKC-pGUS-R1 + TES-pSR1-iSc → AKCS-pGUS-R2 → AK-pGUS (two-step MISSA reactions). See Tables 1 and 2 for nomenclature; TAC-RTL was described previously^13^.

We transformed the helper plasmid pCP20^45^ into the S-IMF strain, resulting in ACS-IMF. We introduced pEVS101^46^ into the ACS-IMF strain by conjugal transfer, resulting in EACS-IMF (E, erythromycin resistance). We subjected overnight cultures of the EACS-IMF strain to heat shock at 42 °C for 1 h and mixed the heat shock-treated culture with an overnight culture of the AK-pGUS strain (600 μl each), washed twice with LB, resuspended in 600 μl LB, and took 180 μl to spread onto LB plates supplemented with the appropriate antibiotics, resulting in the creation of EAKS-pGUS-IMF strain harboring the pGUS-IMF construct.

### Formulas for MISSA reactions for assembly of piABA71

1. AKG-pCB-RTL + TEC-pLC2-olexA-GUS → AKC-piABA71-R1
2. AKC-piABA71-R1 + TEG-pRG2-olexA-NCED3 → AKG-piABA71-R2
3. AKG-piABA71-R2 + TEC-pLC2-olexA-RCAR11 → AKC-piABA71-R3
4. AKC-piABA71-R3 + TEG-pRG2-olexA-LOS5 → AKG-piABA71-R4
5. AKG-piABA71-R4 + TEC-pLC2-2x35Sp-XVE → AKC-piABA71-R5
6. AKC-piABA71-R5 + TEG-pRG2-Hyg → AKG-piABA71-R6
7. AKG-piABA71-R6 + TES-pSL2-2xLB → AKGS-piABA71 → AK-piABA71

See Tables 1 and 2 for nomenclature.

### Formulas for MISSA reactions for assembly of p2x3sgR

1. KG-pCB-RTL2 + TEC-pVLC-2sgR → KC-p2x3sgR-R1
2. KC-p2x3sgR-R1 + TEG-pVRG-2sgR@Sa → KG-p2x3sgR-R2
3. KG-p2x3sgR-R2 + TEC-pVLC-LBH-2sgR@ap → KC-p2x3sgR

See Tables 1 and 2 for nomenclature. Detailed descriptions of creation of the donor and recipient vectors for p2x3sgR, and detailed descriptions of the generation of CRISPR/Cas9 transgenic Arabidopsis and analysis of mutations, are provided in Supplementary Methods S1.

## Additional Information

**How to cite this article**: Zhang, H.-Y. *et al*. MISSA 2.0: an updated synthetic biology toolbox for assembly of orthogonal CRISPR/Cas systems. *Sci. Rep.* **7**, 41993; doi: 10.1038/srep41993 (2017).

**Publisher's note:** Springer Nature remains neutral with regard to jurisdictional claims in published maps and institutional affiliations.

## Supplementary Material

Supplementary Information

Supplementary Dataset 1

Supplementary Dataset 2

Supplementary Dataset 3

## Figures and Tables

**Table 1:** Nomenclature of MISSA donor strains.

Background strain	CTP^a^	Engineered strain^b^	Donor vector	Donor strain^c^
P254D	—	P254D	pLC2-GOI *et al*.	—
P254D	F′DOT-TE	P254D-TE	—	P254D-TE
BW20767	chromosome	BW20767	—	BW20767
P254D	F′DOT-TE	P254D-TE	pLC2-GOI *et al*.	TEC-pLCM2-GOI *et al*.
BW20767	chromosome	BW20767	pLC2-GOI *et al*.	SC-pLCM2-GOI *et al*.

^a^CTP, conjugal transfer protein provider.

^b^The nomenclature of engineered strains is based on the background *E. coli* strain and CTP.

^c^The nomenclature of donor strains harboring donor vectors is mainly based on antibiotic resistances of the donor strain followed by the name of the donor vector. S, spectinomycin resistance conferred by BW20767 chromosome, or donor vectors (pSL- and pSR-). TE, tetracycline and erythromycin resistances conferred by F′DOT-TE. C/G, chloramphenicol/gentamycin resistances conferred by donor vectors (pLC2-, pRG2, pVLC- and pVRG-).

**Table 2:** Nomenclature of MISSA recipient strains.

Background strain	SRP^a^	Engineered strain^b^	Recipient vector	Recipient strain^c^
DH10B	chromosome	DH10B-SRP	—	DH10B-SRP
EPI300	pAH57-Cre-TF	EPI300/SRP	—	EPI300/SRP
DH10B	chromosome	DH10B-SRP	pCB-LTR/RTL	KG-pCB-LTR/RTL
EPI300	pAH57-Cre-TF	EPI300/SRP	pCB-LTR/RTL	AKG-pCB-LTR/RTL
DH10B-RV	—	DH10B-RV	chromosome	—
DH10B-RV	pAH57-Cre-TF	DH10B-RV/SRP	chromosome	AGS-RV

^a^SRP, site-specific recombination protein provider.

^b^The nomenclature of engineered strains is based on the background *E. coli* strain and SRP.

^c^The nomenclature of a recipient strain is mainly based on antibiotic resistances of the recipient strain followed by the name of recipient vector. K/G/A/S, kanamycin/gentamycin/ampicillin/spectinomycin resistances.
