# Supplementary material for: MISSA 2.0: an updated synthetic biology toolbox for assembly of orthogonal CRISPR/Cas systems
Source: Sci Rep. 2017 Feb 3;7:41993. doi: 10.1038/srep41993 (PMC5290471; doi:10.1038/srep41993)
Supplement: Supplementary Information [file srep41993-s1.pdf]

Supplementary information

Table of contents

Table of contents....1
Supplementary Figures 2
Supplementary Figure S1 2
Supplementary Figure S2 3
Supplementary Figure S3 4
Supplementary Figure S4 5
Supplementary Figure S5 6
Supplementary Figure S6 7
Supplementary Figure S7 8
Supplementary Figure S8 9
Supplementary Figure S9 10
Supplementary Figure S10 11
Supplementary Tables....12
Supplementary Table S1: Please see the Excel file....12
Supplementary Table S2: Please see the Excel file....12
Supplementary Table S3....12
Supplementary Table S4....13
Supplementary Methods S1....18
Creation of the donor vectors pLC2-ccdB and pRG2-ccdB....18
Creation of the donor vectors pSL-ccdB and pSR-ccdB....19
Creation of the donor vectors pVLC-ccdB and pVRG-ccdB 19
Creation of the universal functional donor vectors 20
Creation of the donor vectors for the XVE-based inducible expression system 20
Creation of the donor vectors for pABA61....21
Creation of the donor vectors for pTEST51....21
Creation of the donor vectors for pGUS-IMF 21
Creation of the donor vectors for piABA....22
Creation of the recipient binary vectors pCB-RTL/LTR 22
Creation of the *Agrobacterium* helper plasmid pSAH....23
Creation of the host strains ABO, 254D, 203L, and P254D for suicide donor vectors....23
Creation of the engineered strain DH10B-RV and recipient strains....25
Creation of the engineered strain DH10B-SRP and recipient strains 26
Formulas for MISSA Reactions for Assembly of pABA61....27
Formulas for MISSA Reactions for Assembly of pTEST51....27
Creation of the donor and recipient vectors for p2x3sgR....27
Generation of CRISPR/Cas9 transgenic *Arabidopsis* and analysis of mutations 29
References....30
Supplementary File S1: Please See the ZIP file. 31

Supplementary Figures

Supplementary Figure S1

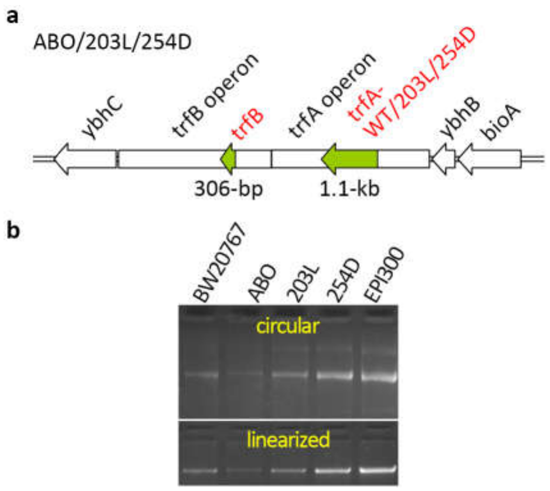

Figure S1. Comparison of copy numbers of the representative RK2-based suicide donor vector pVLC-GUS in five different host strains. (a) Physical maps and structures of the modified genomic regions of the three host strains for the RK2-based suicide donor vectors. **(b)** Comparison of vector concentrations by agarose gel electrophoresis and ethidium bromide–staining. Each of the vectors was extracted from 3.5 ml cultures (OD600 = 1.0), and eluted with 50 µl 10 mM Tris buffer (pH7.5). Five microliters of each vector from the five different strains was loaded with or without linearization with *Hin*dIII.

Supplementary Figure S2

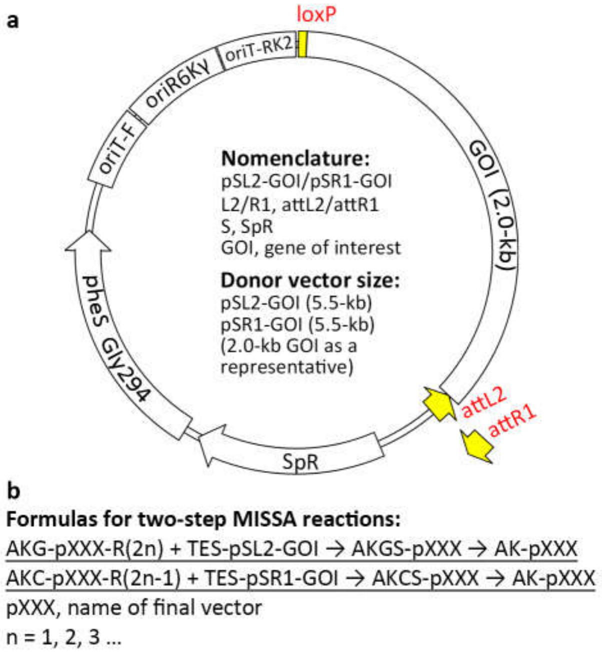

Figure S2. Physical maps and structures of two special donor vectors designed for final round of two-step MISSA reactions. (a) Physical maps and structures of two donor vectors. **(b)** Formulas for two-step MISSA reactions to remove the antibiotic resistance genes from the final recipient vectors.

Supplementary Figure S3

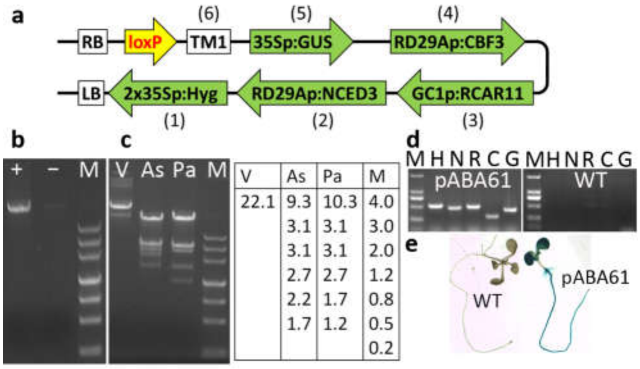

Figure S3. Validation of donor vectors pLC2-/pRG2-, donor strain P254D-TE, recipient vector pCB-LTR, and recipient strain EPI300/SRP. (a) Six rounds of MISSA reactions resulting in production of the final binary vector pABA61. **(b)** The copy number of pABA61 greatly increased when the *E. coli* cells were induced with L-Ara. "+" or "−"indicates whether 0.2% L-Ara was added to the LB broth. M, DNA markers. (c) Restriction enzyme digestion analysis of pABA61. The sizes (kb) of the vector (V), predicted fragments produced by digestion with *Asc*I (As) or *Pac*I (Pa), and DNA markers (M), are indicated. (d) Validation of pABA61 by detecting transgenes in *Arabidopsis*. PCR fragments were amplified from a representative pABA61 transgenic line with a wild-type plant as negative control. H, *Hyg*; N, *NCED3*; R, *RCAR11*; C, *CBF3*; G, *GUS*; M, DNA markers (2.0-kb, 1.0-kb, 750-bp, 500-bp, 250-bp, and 100-bp). (e) GUS staining analysis.

Supplementary Figure S4

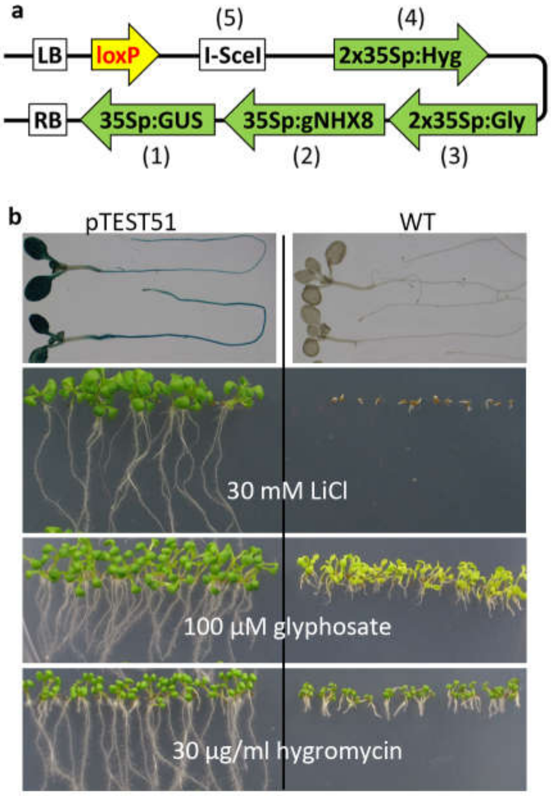

Figure S4. Validation of donor vectors pVLC2-/pVRG2- in donor strain P254D-TE and recipient vector pCB-RTL in recipient strain DH10B-SRP. (a) Five rounds of MISSA reactions resulting in the final binary vector pTEST51. **(b)** Validation of pETST51 by functional verification of transgenes in *Arabidopsis*. T2 plants derived from a representative pTEST51 transgenic T1 line are shown. For GUS staining, T2 seeds from a representative line were sown on MS agar plates containing 25 mg/l hygromycin whereas wild-type (WT) seeds were sown on MS agar plates. The seeds were vernalized at 4°C for 3 days, and then grown under long-day conditions (16 h light/8 h dark) at 22°C for 9 days before GUS staining. Only two representative GUS-stained T2 or WT plants were shown. For Li cation-, glyphosate- or hygromycin-resistance analysis, the seeds were sown on MS agar plates supplemented with 30 mM LiCl, 100 µM glyphosate, or 30 mg/L hygromycin, vernalized at 4°C for 3 days, and grown under long-day conditions (16 h light/8 h dark) at 22°C for 7 days before being photographed.

Supplementary Figure S5

```
Predicted         ..GTGTAAAC.AGGATCCAAGCTTCATGGATAACTTCGTATAGCATACATTATACGAAGTTAT   60
Sequenced_(rc)    TTGTGTAAACCAGG.TCCAAGCTTCATGGATAACTTCGTATAGCATACATTATACGAAGTTAT   62
loxP              .............................ATAACTTCGTATAGCATACATTATACGAAGTTAT   34
I-SceI            ...............................................................    0
attB1             ...............................................................    0

Predicted         GTCGACTAGGGATAACAGGGTAATGGAAGCTTATTAGAATTCGACCTAAAGTCGGCGCGCCAA  123
Sequenced_(rc)    GTCGACTAGGGATAACAGGGTAATGGAAGCTTATTAGAATTCGACCTAAAGTCGGCGCGCCAA  125
loxP              ...............................................................   34
I-SceI            ......TAGGGATAACAGGGTAAT.......................................   18
attB1             ...............................................................    0

Predicted         GCTATCAAACAAGTTTGTACAAAAAAGCAGGCTTTTTAATTAACGACTTTAGGTCCAAGCTTG  186
Sequenced_(rc)    GCTATCAAACAAGTTTGTACAAAAAAGCAGGCTTTTTAATTAACGACTTTAGGTCCAAGCTTG  188
loxP              ...............................................................   34
I-SceI            ...............................................................   18
attB1             ........ACAAGTTTGTACAAAAAAGCAGGCT..............................   25

Predicted         CATGCCTGCAGGTCCCCAGATTAGCCTTTTCAATTTCAGAAAGAATGCTAACCCACAGATGGT  249
Sequenced_(rc)    CATGCCTGCAGGTCCCCAGATTAGCCTTTTCAATTTCAGAAAGAATGCTAACCCACAGATGGT  251
loxP              ...............................................................   34
I-SceI            ...............................................................   18
attB1             ...............................................................   25

Predicted         TAGAGAGGCTTACGCAGCAGGTCTCATCAAGACGATCTACCCGAGCAATAATCTCCAGG....  308
Sequenced_(rc)    TAGAGAGGCT.ACGCAGCAGTTATACAAGGCGGG.............................  284
loxP              ...............................................................   34
I-SceI            ...............................................................   18
attB1             ...............................................................   25
```

Figure S5. Alignment of the predicted sequence and the actual sequence of PCR products sequenced with primer 308R.

Supplementary Figure S6

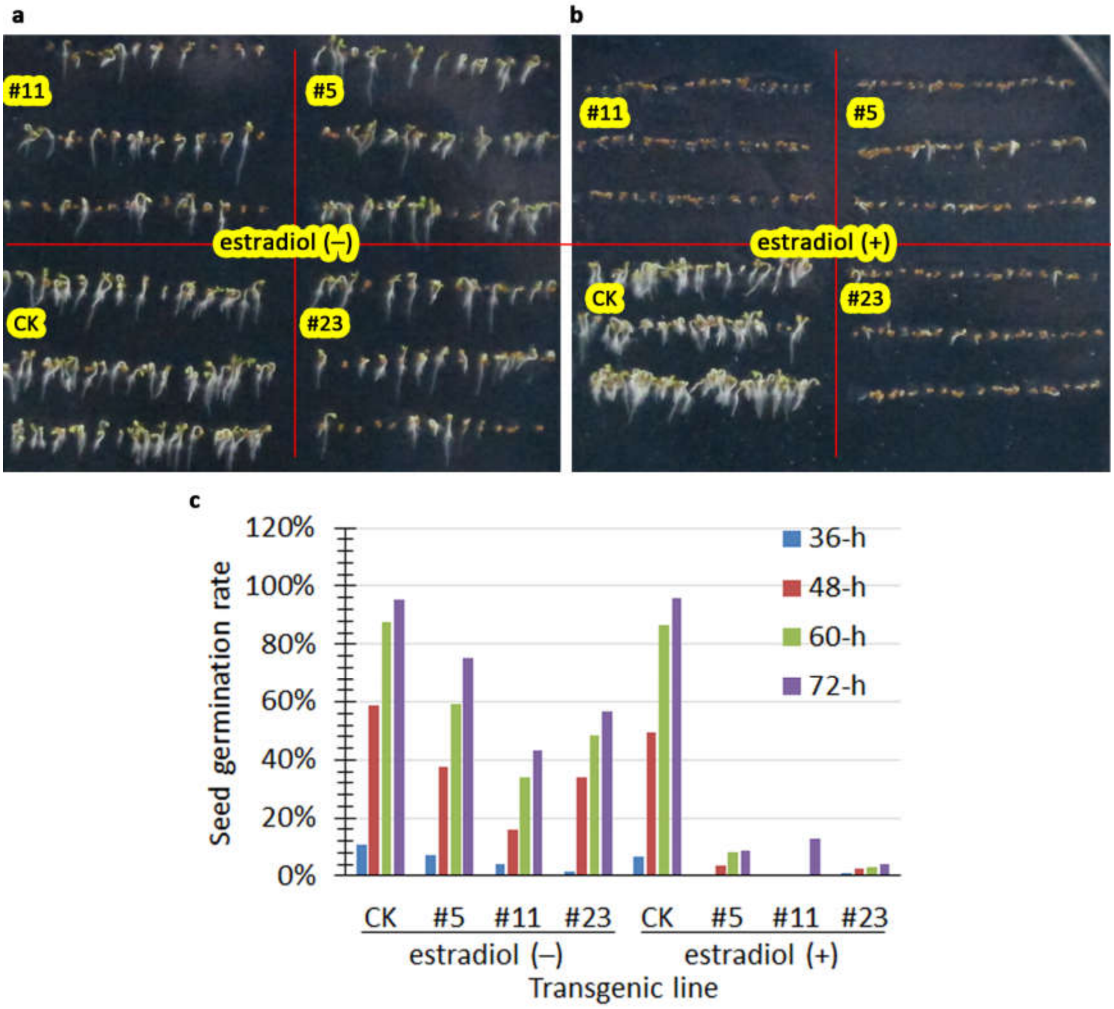

Figure S6. Seed germination of the transgenic lines was seriously affected under estradiol-induced conditions. (a,b) Seed germination analysis of three representative lines under estradiol-induced (+) or non-induced (−) conditions. The T3 seeds were sown on two MS agar plates supplemented with 25 mg/L hygromycin or 25 mg/L hygromycin plus 10 µM α-estradiol, vernalized at 4°C for three days, and grown under long-day conditions (16 h light/8 h dark) at 22°C for 72 h before being photographed. CK, T3 plants derived from a hygromycin-resistant transgenic line. **(c)** Seed germination rate. The germinated seeds grown on the above plates were counted at 36 h, 48 h, 60 h, and 72 h, respectively.

Supplementary Figure S7

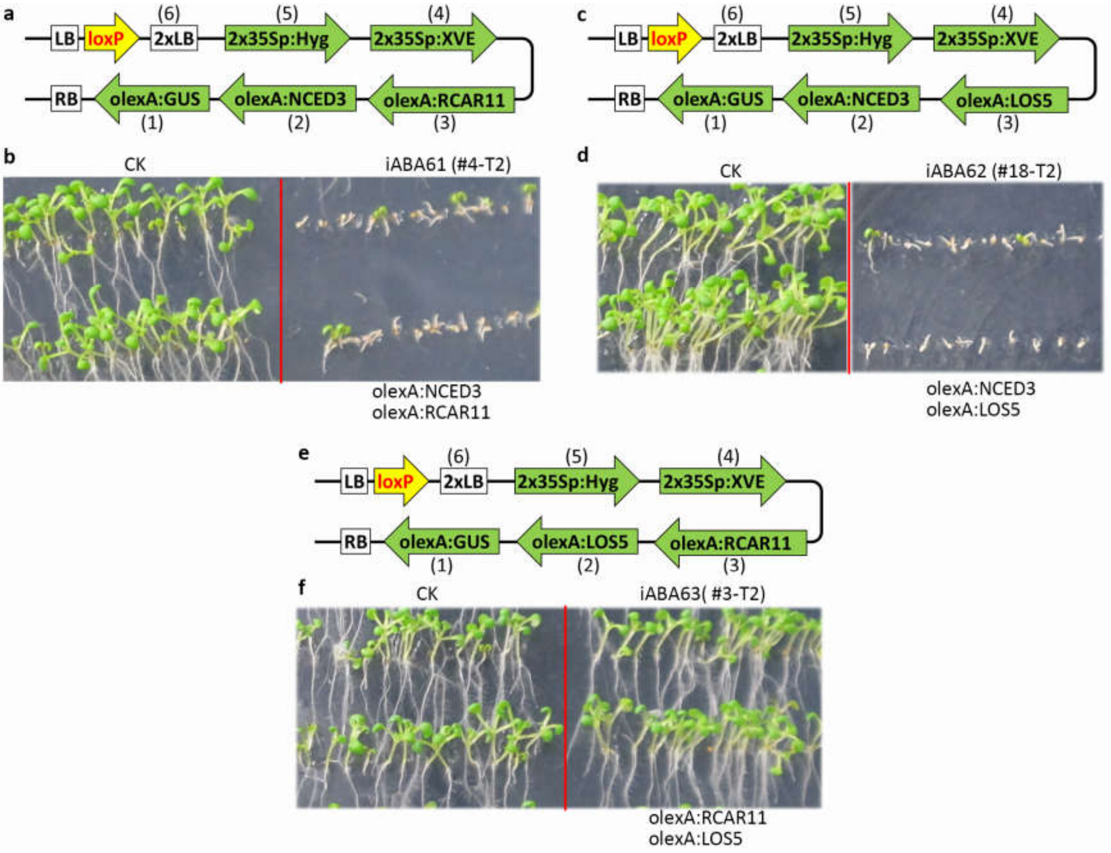

Figure S7. Seed germination and growth analysis of transgenic lines inducibly overexpressing two of the three genes. (a,c,e) Structures of the T-DNA regions of piABA61/62/63, each of which was assembled by 6 rounds of MISSA reactions and represents one of three combinations of two of the three genes. **(b,d,f)** Germination and growth analysis under induced conditions. The T2 seeds from representative T1 lines were sown on MS agar plates supplemented with 25 mg/l hygromycin plus 10 μM α-estradiol, vernalized at 4°C for 3 days, and grown under long-day conditions (16 h light/8 h dark) at 22°C for 7 days before being photographed.

Supplementary Figure S8

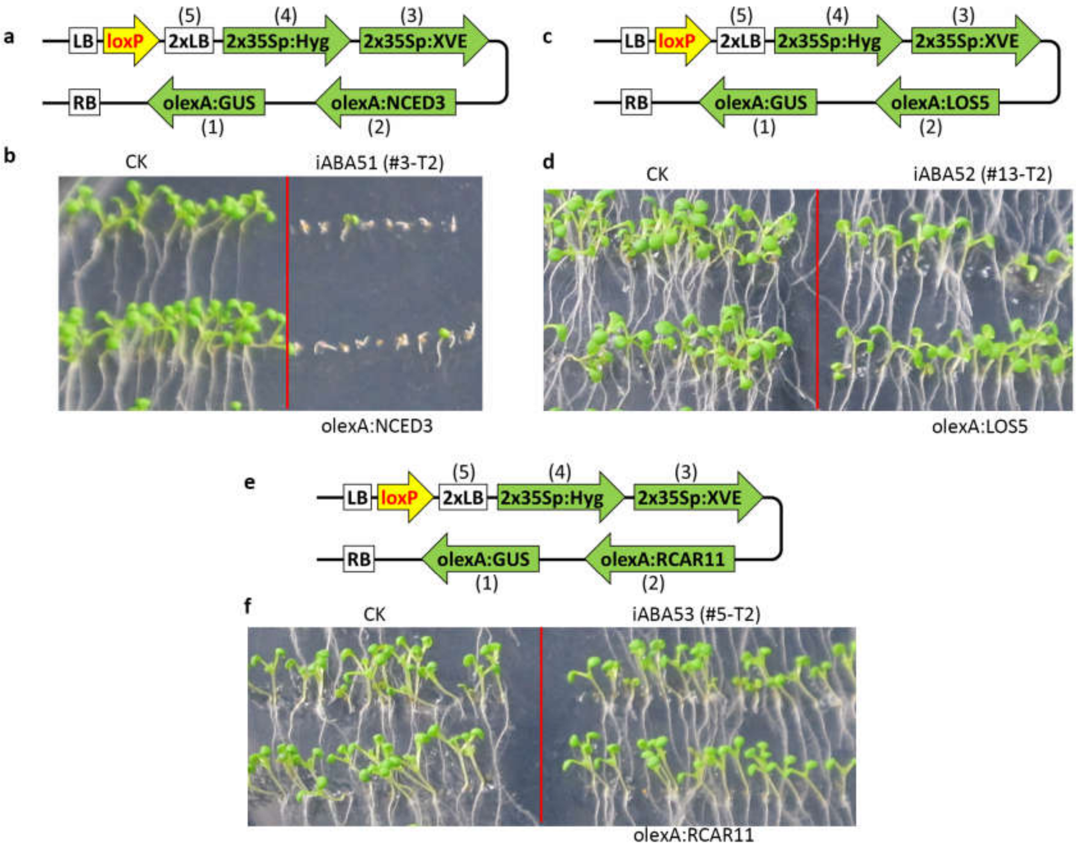

Figure S8. Seed germination and growth analysis of transgenic lines inducibly overexpressing one of the three genes. (a,c,e) Structures of the T-DNA regions of piABA51/52/53, each of which was assembled by 5 rounds of MISSA reactions and inducibly overexpressed one of three genes. **(b,d.f)** Germination and growth analysis under induced conditions. The T2 seeds from representative T1 lines were sown on MS agar plates supplemented with 25 mg/l hygromycin plus 10 µM α-estradiol, vernalized at 4°C for 3 days, and grown under long-day conditions (16 h light/8 h dark) at 22°C for 7 days before being photographed.

Supplementary Figure S9

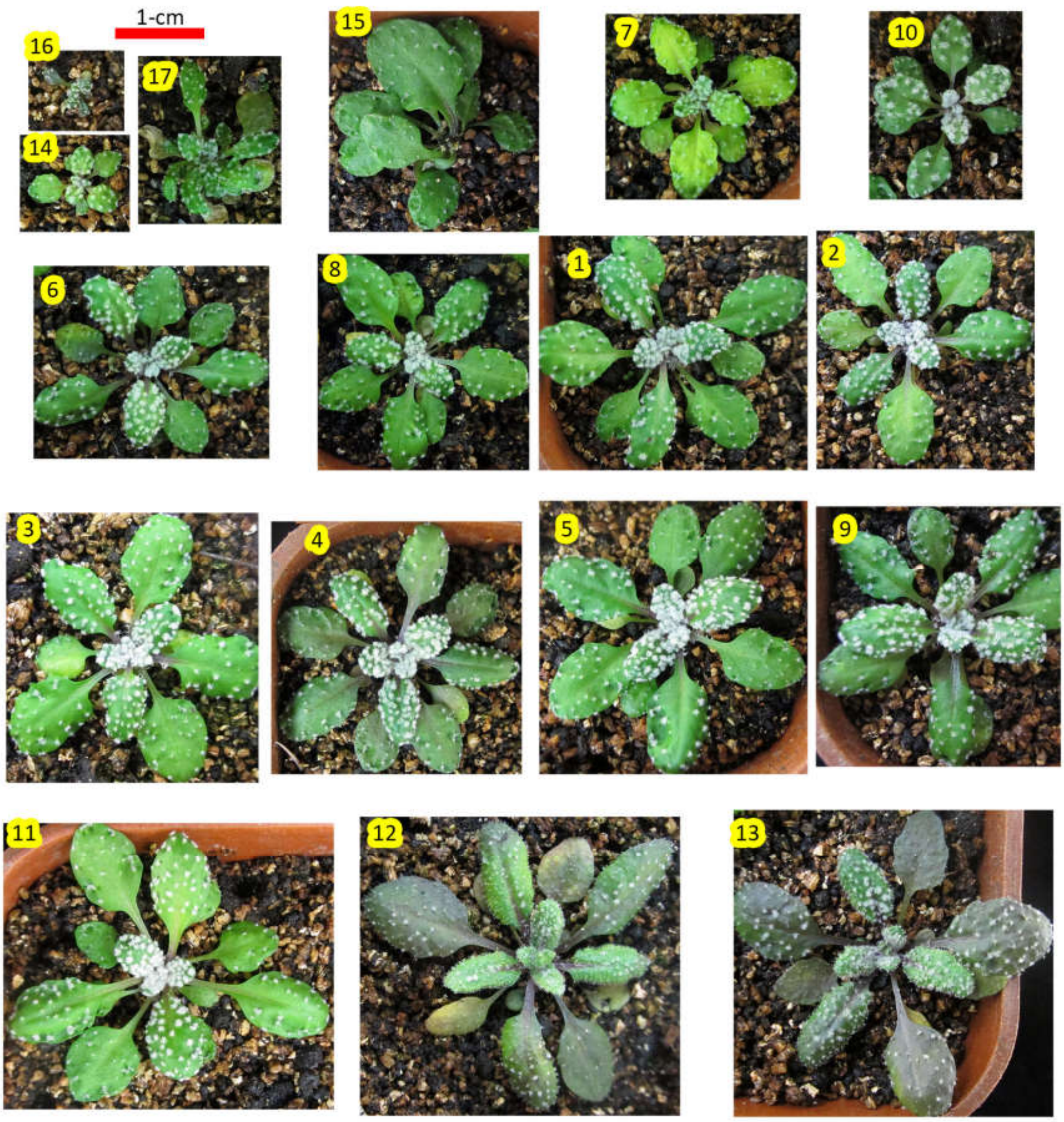

Figure S9. Phenotypes of 17 likely *try cpc* double mutants out of 228 T1 lines. The number of each line corresponds to that in Fig. S10.

Supplementary Figure S10

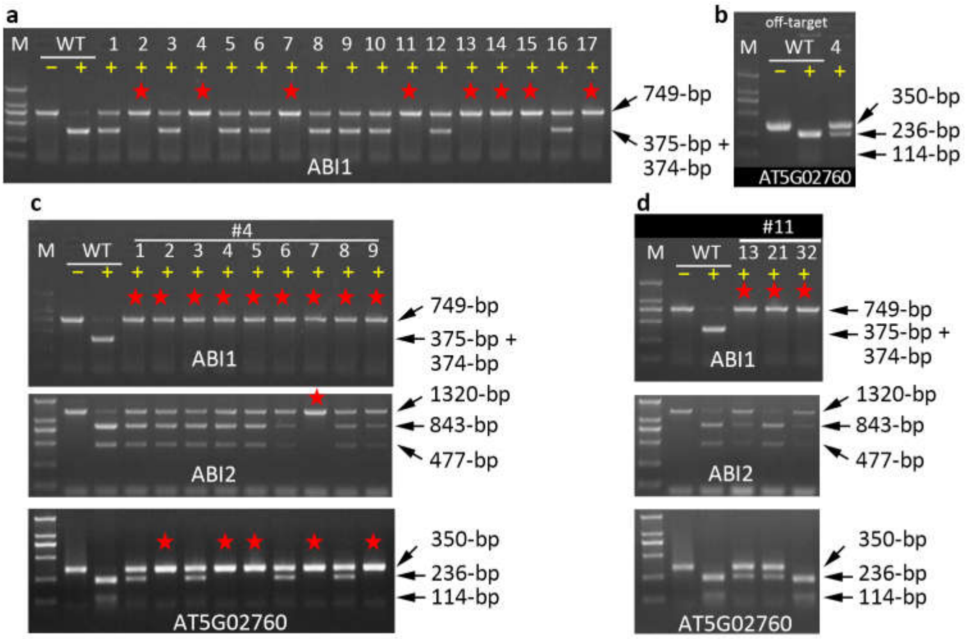

Figure S10. *Nco*I digestion analysis of on-target and off-target mutations induced by sgR-A1&2. (a) *ABI1* mutations of 17 likely *try cpc* double mutants. **(b)** Off-target mutations in AT5G02760. (c) *ABI1*, *ABI2* and *AT5G02760* mutations of 52 T2 plants from #4 T1 line. Only 9 lines of interest are displayed. (d) *ABI1*, *ABI2* and *AT5G02760* mutations of 41 T2 plants from #11 T1 line. Only 3 lines of interest are displayed. The stars indicate homozygous or biallelic mutations.

Supplementary Tables

Supplementary Table S1: Please see the Excel file

Supplementary Table S2: Please see the Excel file

Supplementary Table S3

Table S3. Mutation analysis of 17 T1 plants with clustered leaf trichomes

	**2sgR-3**		**2sgR-2**		**2sgR-1**			
**Line**	**sgR-A2@Sa**	**sgR-H1**	**sgR-T@Sa**	**sgR-C@Sa**	**sgR-A1&2**		**sgR-T&C**	
	**ABI2**	**HAB1**	**TRY**	**CPC**	**ABI1**	**ABI2**	**TRY**	**CPC**
1	ND	+T/+T	BC	ND	CM	ND	BC	BC
2	ND	BC	BC	ND	HB	ND	BC	+A/+C
3	ND	BC	BC	ND	CM	ND	BC	+A/+A
4	ND	BC	+A/+A	ND	HB	ND	BC	+T/+T
5	ND	+T/+T	+C/+T	ND	CM	ND	BC	+A/+A
6	ND	BC	ND	ND	CM	ND	BC	BC
7	ND	-T/-T	+A/+T	ND	HB	ND	BC	BC
8	ND	BC	ND	ND	CM	ND	BC	+A/+C
9	ND	BC	ND	ND	CM	ND	BC	BC
10	ND	BC	ND	ND	CM	ND	BC	BC
11	ND	BC	BC	ND	HB	ND	BC	BC
12	ND	BC	ND	ND	CM	ND	BC	+G/+G
13	ND	BC	ND	ND	HB	ND	BC	+G/+G
14	ND	-T/-T	BC	ND	HB	ND	BC	BC
15	ND	BC	ND	ND	HB	ND	BC	BC
16	ND	BC	+T/+T	ND	CM	ND	BC	BC
17	ND	BC	BC	ND	HB	ND	BC	+A/+T

ND, not detected; HB, homozygous or biallelic mutations; BC, biallelic or chimeric mutations; CM, chimeric mutations. The inserted (+) or deleted (-) nucleotide is denoted. *ABI1* mutations were determined by *Nco*I digestion analysis whereas mutations of the other targets were determined by direct sequencing of PCR products. For homozygous mutants or wild type, we obtained high quality sequencing chromatograms, which means that four kinds of sequencing peaks were well separated. For biallelic mutations with the same number of insertions or deletions at two alleles, we could also obtain high quality sequencing chromatograms except that one or more double-peaks around cleavage sites appeared. For mosaic or biallelic mutations with different number of insertions or deletions at two alleles, we could not obtain high quality sequencing chromatograms behind cleavage sites, which means four kinds of sequencing peaks behind cleavage sites were indistinguishable.

Supplementary Table S4

Table S4. Primers used in this report

Primer name	Primer sequence (5′→3′)
oriTF-SaF	atttgtcgacgctagtataatttaacccactccac
oriTF-CXR	atatcgattaactcgagacactagaagggacgcac
Gen-CNF	aatttatcgatgcggccgcacaccgtggaaacggat
Gen-R2	ttctgcggcgttgtgacaatttacc
pheS-5PF	tgtcatgggtaaaaacggtaaatggctg
pheS-5PR	tccacttccgcagaaggttcggtaaacg
Ap-NcF	atttatccatggttcggggaaatgtgcgc
Ap-XbR	aaatattctagatgacgctcagtggaacg
R6K-NXF	aatatatgcggccgcaactcgagcccatgtcagccgtt
sacB-3F	gaaaatgccgatatcctattggcattgg
lox22-SaR	aattagtcgacataacttcgtataatgtatgct
MISSA-F	attacggtcgactctagcatggatct
CmF3	attacgcattaggcacccccaggc
CmR3	ccgtcctgatagtttggctgtgag
Gen-F2	acaccgtggaaacggatgaaggcac
omEmSF	attgacccaagtaccgccacctaacaattcgttcaagccgagatcggcttcccggcggc
omEmSR	cgccgggaagccgatctcggcttgaacgaattgttaggtggcggtacttgggtcaat
Lox-NcF	aatattaccatggataacttcgtatagcatacatt
RVM21-Xh	atactattctcgagcggataacaatttcacac
oriTRK2-BsF	atattattcatgataggtgggctgcccttc
oriTF-XhR	atattactcgagacactagaagggacgcac
Em7pS-F	gaactaaacccaggaggcagatcatgggtgaagcggtgatcgccgaagt
S-Ater-R	atgagtaaacttggtctgacagttatttgccgactaccttggtgat
S-Ater-F	atcaccaaggtagtcggcaaataactgtcagaccaagtttactcat
Em7pS-R	acttcggcgatcaccgcttcacccatgatctgcctcctgggtttagttc
Ater-BsF	atattattcatgactgtcagaccaagtttactcat
SpR-NhR	attattgctagcttatttgccgactaccttg
pheS-XbF	aattattctagatgggataggctctaagtc
pheS-NcR	ataattccatggttcataatctattcctgcctt
AiScF	attaccctgttatccctattcggggaaatgtgcgc
AiScR	attaccctgttatccctatgacgctcagtggaacg
iSc-SaFR	attattgtcgactccattaccctgttatcc
oriV-XhF	aatattatttaattctcgagctggttgccctcgccgct
oriV-ASR	attatttcttaagttgtcgacctcgaccgggagggttc
oriT-XhF	aatatatctcgagatcaattccgttttccgctgcat
oiSceI-SHEF	tcgactagggataacagggtaatggaagcttattag
oiSceI-SHER	aattctaataagcttccattaccctgttatccctag
o2LB-F1	ttaagtggcaggatatattgtggtgtaaacagtcgagctgttggctgg
o2LB-R1	cgactgtttacaccacaatatatcctgccac

o2LB-F2	ctggtggcaggatatattgtggtgtaaacag
o2LB-R2	aattctgtttacaccacaatatatcctgccaccagccagccaacagct
Ap-SpF	tatttgcatgcttcggggaaatgtgcgc
Ap-KpR	tatttggtacctgacgctcagtggaacg
oAXSNSPF	cgcgccatctagaaatttaaataccatggagagctcattaat
oAXSNSPR	taatgagctctccatggtatttaaatttctagatgg
Ap-PESF	tttacttaattaatagatatcatactagttcggggaaatgtgcgcg
Ap-AKR	ttatttggcgcgccttggtacctgacgctcagtggaacg
2x35S-hEcF	atcttcttatattggctagagcagcttg
2x35S-SpR	tattgactagtggattgtgcgtcatccctt
GC1p-SpF	atttagcatgcattcagtaacccgatgctcctg
GC1p-XbR	attactctagatttcttgagtagtgattttgaagtagtg
RCAR11-XbF	cattattctagatgccttcggagttaacaccag
RCAR11-SaR	atttagagctcacgtcacctgagaaccacttcc
oXb-SaF	ctagagcccgggtaccataccatggatgagct
oXb-SaR	catccatggtatggtacccgggct
Gly-KpF	attatttggtacccctgacaagctgactctagc
Gly-SaR	attcttagagctcatcaggcagccttcgtatc
Ocst-SaF	atttatgagctcctagagtcctgctttaatgagat
Ocst-AsR	attatttggcgcgcctgctgagcctcgacat
oSm-SaF	gggttctcgagttatttaaattaccatggatggtacctagagct
oSm-SaR	ctaggtaccatccatggtaatttaaataactcgagaaccc
Sp-AsF	atatttaggcgcgccaacccagtggacataagc
ST-R	cagacgaacgaagagcgattgagggcttattatgcacgctt
ST-F	aagcgtgcataataagccctcaatcgctcttcgttcgtctg
oriT-PaR	aatattgttaattaagtgtagctctttggcatcgt
oSHEP-F	tcgactcaagcttccttgaattctcttaat
oSHEP-R	taagagaattcaaggaagcttgag
RCAR11-NcF	tataccatggatgccttcggagttaacaccag
RCAR11-SaR	ttattagagctcacgtcacctgagaaccactt
LOS5-hSMF	aaatcccgggattcggcgatggaagcatt
LOS5-SaR	ttctagagctcacgataatacacacaagc
Kan-SAPF	aattcatttaaatcttaagttactgcagaatctctgatgttacattgcac
KT-R	gagacgatgccaaagagctacaccaatatgcctcgtgaagaaggtgttgc
KT-F	gcaacaccttcttcacgaggcatattggtgtagctctttggcatcgtctc
oriT-SaR	attatagtcgacctcaatcgctcttcgttcgtctg
lox-XPF	tattctcgagcacgtgataacttcgtatagcatacatt
Amp-R	agggaataagggcgacacggaaatg
trfA-HiF	atattcaagcttaatcgaaccgcacgacgtag
trfA-XbR	tattctctagataggttaaacccgctccgatc
trfB-XbF	attattctagactgcatgatggtgatagccgt
trfB-EcR	attattgaattcgtgcgcagcagctcgttatc
Sp-EcF	atttattgaattcgaacccagtggacataagc

Sp-AsR	attatttggcgcgccttgggcttattatgcacgctt
pSAH-AsF	atattattggcgcgcctacacaaattgggagatatatcatgc
pSAH-PaR	aattattgttaattaagctatagtgcagtcggcttctgacgttc
Ap-iSceF	attaccctgttatccctattcggggaaatgtgcgc
Ap-iSceR	attaccctgttatccctatgacgctcagtggaacg
iSceI-EcFR	atattattagaattcattaccctgttatcccta
virGE-PaF	atcttgttaattaagaccataggcgatctcctt
virGE-AsR	atattggcgcgcctgattgagccctttcattg
oAiSP-F	ttaagattaccctgttatccctactaat
oAiSP-R	tagtagggataacagggtaatc
oAsiSPs-F	cgcgcatagggataacagggtaatctgca
oAsiSPs-R	gattaccctgttatccctatg
oANXP-F	aattggcgcgccttccatggttccctcgagtgttaattaact
oANXP-R	ctagagttaattaacactcgagggaaccatggaaggcgcgcc
ybhC-EcF	acatgaattctcaacggattcatttttctatttcatagcc
ybhC-NcR	acatttaccatgggaaagcccaatcttcacatcaatcg
bioA-XhF	aatttctcgagtttgcgggcagtatttgcaatcttc
bioA-EHR	atatcgaattcataagctttaagtgataccagatggcatt
trfA-R	ggtacttctcccatatgaatttcgt
trfA254D-5PF	gtctgatcgtgttgggcacctggaat
trfA254D-5PR	gtgaaatccatcgccgtcgcctgtag
trfA203L-5PF	ggtgttggagtacgcgaagcgcac
trfA203L-5PR	tgtagccacaccagttcgtcatcgtc
uidA-iSceF	tccattaccctgttatccctaacgaactgaactggcagactatcc
uidA-iSceR	tccattaccctgttatccctagataatcggctgatgcagtttctcc
iSce-FR	acatccttccattaccctgttatcc
LoxGR2-XhF	aattctcgagtcatggataacttcgtatagcatacatt
ccdB-5R	cgtgtcaataatatcactctgtacatccac
Cm-PaF	atattgttaattaagtggacataagcctcgttcgg
pUC_ori-XhR	acatattctcgaggtaatacggttatccacag
Sp-F	aaatctgaacccagtggacataagc
Sp-R	ttccttgggcttattatgcacgctt
Sp-ApR	atatattgggccctgggcttattatgcacgctt
R6K-AfR	taatattacttaagaattgatccggccacgatg
oFrt-PXF	taatgaagttcctatactttctagagaataggaacttc
oFrt-PXR	tcgagaagttcctattctctagaaagtataggaacttcattaat
oriTF-SpF	atatttgcatgctagtataatttaacccactccac
oriTF-BsR	tattaccatacccatggacactagaagggacgcac
N-PiScF	acactaacttaattaattaccctgttatcccta
N-PiScF0	attaccctgttatccctattcgttcaggactacaactgc
N-ds-HiR	atattcaagcttagccaaggccaatatctaagtaac
Xis-up-EcF	atttatgaattctttcttgcgtgtaattgcggagac
Int-AiScR0	attaccctgttatccctattatcactgttgattctcgct

Int-AiScR	ctatttaggcgcgccattaccctgttatcccta
oHSE-F	agcttactagtagggataacagggtaatg
oHSE-R	aattcattaccctgttatccctactagta
int-PaF	atcttcttaattaatatcaagcagcagaatcatcac
int-HiR	aatacataagcttgttatttgatttcaattttgtcc
T1T2-HiF	acattcaagctttctcggggacgtctaactac
T1T2-SpR	atatctaactagtaaccctggcgcttaagtaac
**Primers for generation of p2x3sgR**	
ApR-BBF	attattgaagacatattgtgagaccttcggggaaatgtgcgc
ApR-BBR	attattgaagacttaaactgagacctgacgctcagtggaacg
U6pt-NBF	tatattaccatggcagtgactggtctcatt
U6pt-NBF0	ccatggcagtgactggtctcatttttttgcaaaattttccagat
U6pt-NBR	atacaatccatggtctcacaatcactacttcgactctagc
AsgR-F	cgttccactgagcgtcaggtct
26t29p-R	gtcaggctgcagtagtttggattaaagaaattgaacgccgaagaac
26t29p-F	gttcttcggcgttcaatttctttaatccaaactactgcagcctgac
29p-R	ccaatctcttagtcgactctacc
oiCeuI-HNF	agcttcgctaccttaggaccgttatagttacgc
oiCeuI-HNR	catggcgtaactataacggtcctaaggtagcga
U6A-BAF	catattacgtctcgaattacctaggacttgccttccgcac
U6A-BEiCR0	ccttaggaccgttatagttacgtattggtttatctcatcg
U6A-BeiCR	acatatacgtctcgaattcgctaccttaggaccgttatag
SPL-BNF	attttattggtctcacatggaacatttctcacaacacgaagtcac
SPL-BXR	acattatggtctcactagatgatgatgatcttcttctcggaactc
mCherry-F0	aagcttacaattgattgacaactaagctggcacaactatatttcc
mCherry-HMF	acatgcaagcttacaattgattgacaacta
mCherry-EAR	attacgaattcaacctaggctcaatacgataatttatttg
U6-ArF	aacattttattacctaggcgacttgccttccgcacaatac
U6-EcR	atttattatgaattctattggtttatctcatcg
oPiCH-F	taattcgctaccttaggaccgttatagttacga
oPiCH-R	agcttcgtaactataacggtcctaaggtagcgaattaat
oEiCA-F	aattcgtaactataacggtcctaaggtagcgaagg
oEiCA-R	cgcgccttcgctaccttaggaccgttatagttacg
Hyg-HSF	acatttacaagcttacaactagtgaggcggtttgcgtatt
Hyg-EcR	atatttgaattcggatctggattttagtactggatt
LB-HiF	catttatataagcttgaccgcggtgatcacaggcagcaac
LB-SpR	cataataactagtaattcagtacattaaaaacgtccgc
U6A-BAF	catattacgtctcgaattacctaggacttgccttccgcac
U6A-BER	aacatatacgtctcgaattctattggtttatctcatcg
DT1-BsF@T&C	atatatggtctcaattgaatatctctctatctcctcgtt
DT1-F0@T&C	tgaatatctctctatctcctcgttttagagctagaaatagc
DT2-R0@A1	aacccatggccgtcgtaaacaccaatctcttagtcgactctac
DT2-BsR@A1	attattggtctcgaaacccatggccgtcgtaaacaccaa

DT1-BsF@T	atatatggtctcaattggaagtgagcagtatcgaatgtt
DT1-F0@Sa@T	tggaagtgagcagtatcgaatgttttagtactctgg
DT2-R0@C	aactggcgacaggttagagactcaatctcttagtcgactctac
DT2-BsR@C	attattggtctcgaaactggcgacaggttagagactcaa
DT1-BsF@A2	atatatggtctcaattggagatagtgaaggagaaacgtt
DT1-F0@Sa@A2	tggagatagtgaaggagaaacgttttagtactctgg
DT2-R0@H1	aacttgaatggaaaccgtgccccaatctcttagtcgactctac
DT2-BsR@H1	attattggtctcgaaacttgaatggaaaccgtgccccaa
GmR-SaF	atattatgtcgacttcggggaaatgtgcgc
GmR-ApR2	atatattgggcccgattatcaaaaaggatcttcacc
RC-iCeuI-hStFR	ccttcgctaccttaggaccgttatagttac
loxGR2-iCeuF	taggaccgttatagttacgtcatggataacttcgtatagcatacatt
loxGR2-iCeuR2	taggaccgttatagttacgttaagaattatcgaaccactttgt
RBGK-BsF	aatatatggtctcacgtctcagaagccgactgcactat
RBGK-BsR	aatatatggtctcaatacggtctgacgctcagtggaac
oriV-BsF	atatatggtctcagtattaccgctagatagctggtt
oriV-BbR	atttattgaagacattagaccatgactcgaccg
oriT-BsF	attattggtctcatcgcgacctcaatcgctcttcgttcgt
oriT-BbR	atatattgaagacattctaggtgtagctctttggcatcgt
VS1-BsF22	aaatactggtctcagcgagtcagaggttttcaccgtcatc
VS1-BsR22	atttattggtctcagacgttcagtgcagccgtcttctga
**Primers for on/off-target mutation analysis**	
TRY-5U-F	ttcgctctataaactctcatctctcacg
TRY-3U-R	acctaaccgcatggattaaagttgattacc
CPC-5U-F	gtttcgtgtcttcagattagttcgatgt
CPC-3Uds-R	gagctacctcgttgacccatatcgt
ABI2-IDF0	gtcgctgttccattcagaccatt
ABI2-IDR0	cattacatcccaaagaccatcac
ABI1-IDF0	tcatcatgctctgtttctgggtcac
ABI1-IDR0	tatggtcaacggataatggaagtgc
HAB1-IDF	tgttgtggaggataatagtgctgtc
HAB1-IDR	gcctaccctctcctgtattcctctt
AT5G02760-F	atggcaatgatccaagccaacagtg
AT5G02760-R	gtaggccattgcttcgtcactgtct
AT2G25070-F	aagctcagatttggtttatcgtctatgc
AT2G25070-R	acgtctccagttttatacgcttcattac
AT3G17090-F	ggcacgtctgtgataattccgaagc
AT3G17090-R	acctaactttgttgaccaatcaccatct

Supplementary Methods S1

Creation of the donor vectors pLC2-ccdB and pRG2-ccdB

We generated *Sal*I and *Xho*I-*Cla*I-flanked oriTF fragment by PCR amplification from pMAGIC1[1] with primers oriTF-SaF/-CXR. We purified the PCR fragment and inserted it into pGWC[2] by TA cloning, resulting in pGWC-oriTF. We generated *Cla*I-*Not*I-GmR-*Sac*II fragment by PCR amplification from pGWG with primers Gen-CNF/Gen-R2. We purified the PCR fragment, digested it with *Cla*I and *Sac*II, and inserted it into the *Cla*I and *Sac*II sites of pGWC-oriTF to replace the *CmR* fragment, resulting in pG-oriTF. We inserted the *pheS Gly294* fragment between the *Sal*I and *Cla*I sites of pML378[1] into the *Xho*I and *Cla*I sites of pG-oriTF, resulting in pG-oriTF-pheS. We disrupted the *Pst*I and *Aat*II sites of *pheS Gly294* by site-directed mutation with primers pheS-5PF/R phosphorylated at their 5'-end, resulting in pG-oriTF-pheSm.

We generated *Nco*I and *Xba*I-flanked *ApR* frament by PCR amplifcation from pUC18 with primers Ap-NcF/-XbR. We purifed the PCR fragment, digested it with *Nco*I and *Xba*I, and inserted it into the *Bsp*HI and *Nhe*I sites of pLC-ccdB[3], resulting in pLC-Amp. We replaced the *sacB* fragment of pLC-Amp with the *Xho*I site by PCR amplification with primers R6K-NXF/sacB-3F. We purified the PCR fragment, digested it with *Not*I, and allowed it to self-ligate, resulting in pLM2-DsB. We replaced the *CmR* fragment of pLM2-DsB with the *Sal*I site by PCR amplification with primers MISSA-F/lox22-SaR. We purified the PCR fragment, digested it with *Sal*I, and allowed it to self-ligate, resulting in pLM2-DsB2.

We inserted the *Sal*I-*Not*I fragment of oriTF-pheSm from pG-oriTF-pheSm into the *Xho*I and *Not*I sites of pLM2-DsB2, resulting in pLM2BB. We obtained *CmR* gene by PCR amplification from pLC-ccdB[3] with primers CmF3/R3, we purified the PCR fragment, and ligated with *Sal*I-digested and T4 pol blunted pLM2BB, resulting in pLC2-A. We inserted the *Apa*I-*Pst*I fragment of pLACB[3] into the *Apa*I and *Pst*I sites of pLC2-A, resulting in pLC2-ccdB. We obtained *GmR* gene by PCR amplification from pGWG[2] with primers Gen-F2/R2, we purified the PCR fragment, and ligated with *Sal*I-digested and T4 pol blunted pLM2BB, resulting in pLG2-A. We disrupted the *Eco*RV and *Sac*II sites of pLG2-A by inserting a short insert generated by annealing two oligos omEmSF/R into the two sites, resulting in pLG2-A2. We inserted the *Apa*I-*Pst*I fragment of pRACB[3] into the *Apa*I and *Pst*I sites of pLG2-A2, resulting in pRG2-ccdB.

Creation of the donor vectors pSL-ccdB and pSR-ccdB

We generated two PCR fragments from pLM2BB: one was flanked by *Nco*I and *Xho*I introduced by primers Lox-NcF/RVM21-Xh, the other was flanked by *Bsp*HI and *Xho*I introduced by primers oriTRK2-BsF/oriTF-XhR. We purifed the two PCR fragments, digested them with *Nco*I/*Xho*I and *Bsp*HI/*Xho*I, respectively. We ligated the two digested fragments, resulting in pLM2BB-DOP.
We obtained *Nco*I-flanked Em7p:*GmR* with 94-bp terminator from *ApR* gene by gene synthesis, we inserted the *Nco*I fragment into the two *Bsp*HI sites of pUC18 to replace *ApR* gene, resulting in pUC-Em7G. We replaced the *GmR* ORF of pUC-Em7G with *SpR* ORF, resulting in pUC-Em7S. We did this by overlaping PCR with two PCR fragments obtained by PCR amplifcation from pUC-Em7G and pMDC150[4], with primers S-Ater-F/Em7pS-R and Em7pS-F/S-Ater-R, respectively. We introduced *Xba*I and *Bsp*HI sites at the downstream of *SpR* ORF in pUC-Em7S by PCR amplifcation with primers Ater-BsF/SpR-NhR, we amplified the *pheS Gly294* ORF with primers pheS-XbF/-NcR from pRG2-ccdB. We purifed the two PCR fragments, and digested them with *Bsp*HI/*Nhe*I and *Nco*I/*Xba*I, respectively. We purified the digested fragments and allowed them to ligate with each other, resulting in pUC-Em7SP.
We generated *Sal*I and I-SceI-flanked Em7p-SpR-pheS fragment by PCR amplification from pUC-Em7SP with three primers AiScF/R/iSc-SaFR. We purified the PCR fragment, digested it with *Sal*I and inserted it into the *Xho*I site of pLM2BB-DOP, resulting in pSLM2BB. We inserted the *Apa*I-*Pst*I fragment of pLACB[3] into the *Apa*I and *Pst*I sites of pSLM2BB, resulting in pSL-ccdB. We inserted the *Apa*I-*Pst*I fragment of pRACB[3] into the *Apa*I and *Pst*I sites of pSLM2BB, resulting in pSR-ccdB.

Creation of the donor vectors pVLC-ccdB and pVRG-ccdB

We generated *Xho*I and *Afl*II-*Sal*I-flanked oriV by PCR amplification from pCC1BAC (Epicentre Biotechnologies) with primers oriV-XhF/-ASR, we purified the PCR fragment, digested it with *Xho*I and *Afl*II, and inserted it into the *Sal*I and *Afl*II sites of pG-oriTF-pheSm, resulting in pG-oriV-TF-pheS. We generated the *Xho*I and *Not*I-flanked fragment by PCR amplification from pLC2-A with primers oriT-XhF/SacB-3F, we purified the fragment, and digested it with *Xho*I and *Not*I. We allowed the digested PCR fragment to ligate with the oriV-oriTF-pheS fragment cut out from pG-oriV-TF-pheS with *Sal*I and *Not*I, resulting in pVLC-A. We generated pVLG-A in the same manner except that we obtained the PCR fragment from pLG2-A. We inserted the *Apa*I-*Pst*I fragment of pLACB[3] into the *Apa*I and *Pst*I sites of pVLC-A, resulting

in pVLC-ccdB. We inserted the *Apa*I-*Pst*I fragment of pRACB[3] into the *Apa*I and *Pst*I sites of pVLG-A, resulting in pVRG-ccdB.

Creation of the universal functional donor vectors

To generate pLC2-/pRG2-/pVLC-/pVRG-GUS, we inserted the *Hin*dIII-*Eco*RI fragment of *GUS* gene from pBI121 into the *Hin*dIII and *Eco*RI sites of pLC2-/pRG2-/pVLC-/pVRG-ccdB to replace the *ccdB* fragment, resulting in the generation of the corresponding vectors. To generate pLC2-/pRG2-/pVLC-/pVRG-Hyg, we inserted the *Hin*dIII-*Eco*RI fragment of *Hyg* cassette from pL-Hyg into the *Hin*dIII and *Eco*RI sites of pLC2-/pRG2-/pVLC-/pVRG-ccdB to replace the *ccdB* fragment, resulting in the generation of the corresponding vectors. To generate pSL2-/pSR1-iSc, we replaed the *Sal*I-*Eco*RI fragment of pSL-/pSR-ccdB with an insert produced by annealing two oligos oiSceI-SHEF/R. To generate pSL2-/pSR1-2LB, we replaced the *Afl*II-*Eco*RI fragment of pSL-/pSR-ccdB with two inserts with each produced by annealing two oligos o2LB-F1/R1 or two oligos o2LB-F2/R2 (Table S1).

Creation of the donor vectors for the XVE-based inducible expression system

To generate pLC2-/pRG2-olexA, we digested pLC2-/pRG2-ccdB with *Pac*I and *Asc*I, and blunted the fragments with T4 DNA polymerase, and ligated them with an *ApR* fragment obtained by PCR amplification from pUC18 with primer pair Ap-SpF/-KpR, resulting in pLC2-/pRG2-A2. We replaced the *Asc*I-*Pac*I fragment of pMDC221[4] with an insert produced by annealing two oligos oAXSNSPF/R, resulting pMDC221-del. We replaced the *Sph*I-*Kpn*I fragment of *ApR* in pLC2-/pRG2-A2 with the 911-bp *Sph*I-*Kpn*I fragment of *olexA* cassette from pMDC221-del, resulting in pLC2-/pRG2-olexA. We replaced the *Sph*I-*Xba*I fragment of 35S promoter in pLC2-/pRG2-GUS with the *Sph*I-*Xba*I fragment of *olexA* operator from pLC2-olexA, resulting in pLC2-/pRG2-olexA-GUS.

To generate pLC2-/pRG2-XVE, we obtained the *ApR* PCR fragment by PCR amplification from pUC18 with primer pair Ap-PESF/-AKR. We purified the PCR products, digested them with *Pac*I and *Asc*I, and ligated them with *Pac*I and *Asc*I-digested pLC2-ccdB, resulting in pLC2-A3. We replaced the *Spe*I-*Kpn*I fragment of *ApR* in pLC2-A3 with the 2.0-kb *Spe*I-*Kpn*I fragment of *XVE* from pMDC150[4], resulting in pLC2-XVE-P0. We obtained the double 35S enhancer by PCR amplification from pMDC99[5] with primer pair 2x35S-hEcF/SpR. We purified the PCR products, digested them with *Spe*I, and ligated them with *Eco*RV and *Spe*I-digested pLC2-XVE-P0, resulting in pLC2-XVE. We replaced the *Pac*I-*Asc*I fragment of *ccdB* in pRG2-ccdB with the *Pac*I-*Asc*I fragment of *XVE* cassette from pLC2-XVE, resulting in pRG2-XVE.

Creation of the donor vectors for pABA61

To generate pABA61, we constructed donors including pLC2-RCAR11, pRG2-CBF3/NCED3, and pSR-TM1. We obtained the *Arabidopsis* guard cell promoter *GC1*[6] by PCR amplification from *Arabidopsis* genomic DNA with primer pair GC1p-SpF/XbR. We purified the PCR products, digested them with *Sph*I and *Xba*I, and ligated them with *Sph*I and *Xba*I-digested pLC2-GUS, resulting in pLC2-GC1p. We obtained the *Arabidopsis* ABA receptor gene *RCAR11*[7,8] by PCR amplification from *Arabidopsis* genomic DNA with primer pair RCAR11-XbF/SaR. We purified the PCR products, digested them with *Xba*I and *Sac*I, and ligated them with *Xba*I and *Sac*I-digested pLC2-GC1p, resulting in pLC2-RCAR11. We inserted the *Hin*dIII-*Eco*RI fragments of *CBF3*/*DREB1A*[9,10] and *NCED3*[11] cassettes from pL-CBF3 and pR-NCED3 into the *Hin*dIII-*Eco*RI sites of pRG2-ccdB, respectively, resulting in pRG2-CBF3/NCED3. We inserted the *Hin*dIII-*Eco*RI fragment from pL-TM1[3] into the *Hin*dIII-*Eco*RI sites of pSR-ccdB, resulting in pSR-TM1.

Creation of the donor vectors for pTEST51

To generate pTEST51, we constructed donors including pVLC-Gly and pVRG-gNHX8. We replaced the *Xba*I-*Sac*I fragment of *CmR-ccdB* cassette in pMDC32[5] with an insert produced by annealing two oligos oXb-SaF/R, resulting in pMDC32-del. We inserted the *Hin*dIII-*Eco*RI fragment of 2x35Sp cassette into the *Hin*dIII and *Eco*RI sites of pVLC-ccdB, resulting in pVLC-2x35S. We obtained *CTP2-CP4EPSPS* fusion gene harboring an intron from maize *HSP70* by PCR amplification from glyphosate-resistance transgenic maize (Monsanto) genomic DNA with primer pair Gly-KpF/-SaR. We purified the PCR products, digested them with *Kpn*I and *Sac*I, and ligated them with *Kpn*I and *Sac*I-digested pVLC-2x35S, resulting in pVLC-Gly-P1. We replaced the *Sac*I-*Asc*I fragment of the *nos* terminator in pVLC-Gly-P1 with *ocs* terminator obtained by PCR amplification from pR-tOcs[3] with primer pair Ocst-SaF/-AsR, resulting in pVLC-Gly. We replaced the *Xba*I-*Sac*I fragment of *GUS* gene in pVRG-GUS with an insert produced by annealing two oligos oSm-SaF/R, resulting in pVRG-35S. We inserted the 4.3-kb *Xba*I-*Kpn*I fragment of genomic *NHX8* sequence from pBI121-gNHX8[12] into the *Xba*I and *Kpn*I sites of pVRG-35S, resulting in pVRG-gNHX8.

Creation of the donor vectors for pGUS-IMF

To generate pGUS-IMF, we constructed donors including pRG2-zCre and pL2-Hyg-ST. We obtained maize codon-optimized *Cre* gene (*zCre*) harboring an intron via gene synthesis conducted by GenScript (Nanjing). We inserted the *Xba*I-*Sac*I fragment of

zCre into the *Xba*I and *Sac*I sites of pRG2-olexA, resulting in pRG2-zCre. We obtained *SpR* and *oriT* by PCR amplification from pPZP200[13] and pL-oriT[3], with primer pairs Sp-AsF/ST-R and ST-F/oriT-PaR, respectively. We mixed the two PCR fragments and performed fusion PCR, with the mixture as templates, and Sp-AsF/oriT-PaR as primer pair. We purified the PCR products, digested them with *Asc*I and *Pac*I, and ligated them with *Asc*I and *Pac*I-digested pLM2-A, resulting in pL-ST. We replaced the *Sal*I-*Pac*I fragment of pL-ST with an insert produced by annealing oligos oSHEP-F/R, resulting in pL2-ST. We inserted the *Hin*dIII-*Eco*RI fragment of *Hyg* cassette from pRG2-Hyg into the *Hin*dIII and *Eco*RI sites of pL2-ST, resulting in pL2-Hyg-ST. See above for the construction of pLC2-XVE/GUS, and pSR1-iSc.

Creation of the donor vectors for piABA

To generate piABA71, piABA61/62/63, piABA51/52/53, we constructed donors including pRG2-olexA-NCED3, pLC2-/pRG2-olexA-RCAR11, pLC2-/pRG2-olexA-LOS5. We inserted the *Nco*I-*Sac*I fragment of *NCED3* in pR-NCED3[3] into the *Nco*I and *Sac*I sites of pRG2-olexA, resulting in pRG2-olexA-NCED3. We obtained *RCAR11* by PCR amplification from *Arabidopsis* genomic DNA with primer pair RCAR11-NcF/-SaR. We purified the PCR products, digested them with *Nco*I and *Sac*I, and ligated them with *Nco*I and *Sac*I-digested pLC2-/pRG2-olexA, resulting in pLC2-/pRG2-olexA-RCAR11. We obtained *LOS5* by PCR amplification from pR-LOS5[3] with primer pair LOS5-hSMF/-SaR. We purified the PCR products, digested them with *Sac*I, and ligated them with *Swa*I and *Sac*I-digested pLC2-/pRG2-olexA, resulting in pLC2-/pRG2-olexA-LOS5.

Creation of the recipient binary vectors pCB-RTL/LTR

To generate two recipient binary vectors pCB-RTL/LTR, we obtained *KmR* and *oriT* by PCR amplification from TAC-RTL[3] with primer pair Kan-SAPF/KT-R, and from pL-oriT[3] with primers KT-F/oriT-SaR, respectively. We mixed the two PCR fragments and performed fusion PCR with the mixture as templates and Kan-SAPF/oriT-SaR as primer pair. We purified the fused PCR products, and ligated them with *Afl*II and *Sac*II-digested and T4 DNA polymerase-blunted pGWC[2], resulting in pKT. We inserted the *Afl*II-*Pst*I fragment of LB-RB in pGL[14] into the *Afl*II and *Pst*I sites of pKT, resulting in pKT-LR. We obtained loxP-GmR-attR2 cassette by PCR amplification from pLG2-A with primer pair lox-XPF/Amp-R. We purified the PCR products, digested them with *Hin*dIII and *Pml*I, and ligated them with *Hin*dIII and *Pml*I-digested pKT-LR, resulting in pKTG-LTR. In addition, we ligated the *Hin*dIII and *Pml*I-digested PCR products with *Hin*dIII and *Hpa*I-digested pKT-LR, resulting in pKTG-RTL. We digested pKTG-LTR with *Hpa*I and *Pac*I, blunted them with T4

DNA polymerase, and re-ligated them, resulting in pKTG-LTR-del. We digested pKTG-RTL with *Pac*I and *Pml*I, blunted them with T4 DNA polymerase, and re-ligated them, resulting in pKTG-RTL-del. We inserted the *Swa*I-*Sal*I large fragment of pKTG-LTR-del or pKTG-RTL-del into the *Hpa*I and *Xho*I sites of pCC1-BAC (Epicentre Biotechnologies), resulting in pCB-LTR/RTL.

Creation of the *Agrobacterium* helper plasmid pSAH

To generate pSAH, we obtained the *trfA* and *trfB* fragments by PCR amplification from *E. coli* strain BW20676[15] genomic DNA with primer pairs trfA-HiF/-XbR, and trfB-XbF/-EcR, respectively. We mixed the two PCR fragments, purified the PCR products, digested them with *Hin*dIII/*Xba*I/*Eco*RI, and ligated them with *Hin*dIII and *Eco*RI-digested pLC2-ccdB, resulting in pLC2-trfAB. We obtained the *SpR* fragment by PCR amplification from pPZP200[13] with primer pair Sp-EcF/AsR. We purified the PCR products, digested them with *Eco*RI and *Asc*I, and inserted them into the *Eco*RI and *Asc*I sites of pLC2-trfAB, resulting in pLC2-trfAB-S. We obtained the fragment harboring pVS1-origin and pUC-origin for the plasmid replication in *Agrobacterium* and *E. coli*, respectively, by PCR amplification from pPZP200[13] with primer pair pSAH-AsF/PaR. We purified the PCR products, digested them with *Asc*I and *Pac*I, and ligated them with the *Asc*I and *Pac*I-digested pLC2-trfAB-S, resulting in pSAH-trfAB. We obtained the *ApR* fragment flanked by *Eco*RI and I-SceI from pUC18 with three primer mixture Ap-iSceF/R/iSce-EcFR. We purified the PCR products, digested them with *Eco*RI, and inserted them into the *Eco*RI site of pSAH-trfAB, resulting in pSAH-trfAB-A. We obtained the *virG* and *virE* fragment by PCR amplification from pCH32[16] with primer pair virGE-PaF/AsR. We purified the PCR products, digested them with *Asc*I and *Pac*I, and inserted into the *Asc*I and *Pac*I sites of pRG2-ccdB, resulting in pRG2-virGE. We replaced the *Afl*II-*Pac*I region of pRG2-virGE with an insert produced by annealing two oligos oAiSP-F/R, resulting in pVirGE-iSc1. We replaced the *Asc*I-*Pst*I region of pVirGE-iSc1 with an insert produced by annealing two oligos oAsiSPs-F/R, resulting in pVirGE-iSc2. We inserted the I-SceI fragment of *virG-virE* in pVirGE-iSc2 into the two I-SceI sites of pSAH-trfAB-A, resulting in pSAH.

Creation of the host strains ABO, 254D, 203L, and P254D for suicide donor vectors

We replaced the *Eco*RI-*Spe*I region of pDOC-K[17] with an insert produced by annealing two oligos oANXP-F/R, resulting in pDOC-K-del. We obtained *bioA* and *ybhC* fragments by PCR amplification from *E. coli* strain DH10B genomic DNA with primer pairs bioA-XhF/-EHR and ybhC-EcF/-NcR, respectively. We purified the PCR

products, digested the purified *bioA* and *ybhC* PCR products with *Eco*RI/*Xho*I, and *Eco*RI/*Nco*I, respectively. We purified the two digested PCR fragments, and ligated the two fragments with *Nco*I and *Xho*I-digested pDOC-K-del, resulting in pDOC-YB. We inserted the *Hin*dIII-*Eco*RI fragment of *trfA*-*trfB* from pLC-trfAB into the *Hin*dIII and *Eco*RI sites of pDOC-YB, resulting in pDOC-YB-trfAB. We co-transformed pDOC-YB-trfAB and pACBSCE[17] into the *E. coli* strain SW106[18]. We conducted Gene Doctoring experiments according to the protocol[17]. We incubated the agar plates at 42°C to kill the background cells, resulting in the creation of engineered *E. coli* strain ABO, in which 6.4-kb *trfA*-*trfB* was integrated into the chromosome. We obtained the fragment harboring partial *trfA* sequence from pLC2-trfAB with primer pair trfA-HiF/trfA-R. We purified the PCR products and cloned the fragment into the blunt-end cloning vector pCBC, resulting in pCBC-trfA. We introduced the 254D mutation into the sequence by PCR amplification of the whole sequence of pCBC-trfA with primer pair trfA254D-5PF/-5PR phosphorylated at their 5'-end. We purified the PCR products and re-ligated them, resulting in pCBC-trfA254D. In the same manner, we obtained pCBC-trfA203L harboring the 203L mutation introduced with primer pair trfA203L-5PF/R. We replaced the *Hin*dIII-*Nde*I region of pDOC-YB-trfAB with the *Hin*dIII-*Nde*I fragment of pCBC-trfA254D/203L, respectively, resulting in pDOC-YB-trfA254D/230L. In the same manner as we generated *E. coli* strain ABO, we generated the other two strains 254D and 203L.

To generate P254D, we obtained *pir* mutant gene by PCR amplification from *E. coli* strain BW23474[15] genomic DNA with primer pair uidA-iSceF/R. We purified the PCR products and cloned the fragment into the blunt-end cloning vector pCBC, resulting in pCBC-pir106L. We found that the *pir116* named previously was actually *pir106L* mutation by sequencing analysis; therefore we re-named the *pir* copy-up mutant gene *pir106L*. We obtained *ApR* fragment flanked by I-SceI from pSAH-trfAB-A with a single primer iSce-FR. We purified the PCR products, and ligated them with *Nae*I-digested pACBSR[17], resulting pACBSR-A. We inserted the I-SceI fragment of pCBC-pir106L into the two I-SceI sites of pACBSR-A, resulting in pACBSR-pir106L, which we used thereafter for Gene Doctoring experiments instead of two plasmids pDOC and pACBSCE. We co-transformed the pACBSR-pir106L and pRG2-A2 into the *E. coli* strain 254D. We conducted Gene Doctoring experiments according to the protocol[17] modified based on one-plasmid strategy. We selected the engineered strain P254D harboring pRG2-A2 on agar plates supplemented with ampicillin and gentamycin. We purged the plasmid pRG2-A2 from the strain by selecting single colonies on Cl-Phe agar plates (0.5% w/v yeast extract, 1% w/v NaCl, 0.4% w/v glycerol, 2% w/v agar, 10 mM D,L-p-Cl-Phe), resulting in plasmid-free *E. coli* strain P254D.

Creation of the engineered strain DH10B-RV and recipient strains

We cut out Frt-KmR-Frt fragment from pDOC-H[17] with *Sma*I and *Sci*I, and we ligated the fragment with the *Mlu*I-digested and T4 Pol-blunted pCBC-pir106L, resulting in pCBC-FKF, in which *KmR* was flanked by I-SceI-5'uidA-Frt and Frt-3'uidA-I-SceI. We cut out the I-SceI fragment of *KmR* from pCBC-FKF with I-SceI, and ligated the fragment with I-SceI-digested pACBSR-A to replace *ApR*, resulting in pACBSR-FKF. We transformed the pACBSR-FKF into the *E. coli* strain DH10B and conducted Gene Doctoring experiments according to the protocol[17], resulting in DH10B-FKF. In this experiment, we used one-plasmid (pACBSR-FKF) strategy to replace two-plasmid strategy (pDOC and pACBSCE). We transformed pCP20 into DH10B-FKF, and allowed Flp/Frt-mediated SSR reaction to occur according to the protocol, resulting in DH10B-Frt.

We generated the loxP-GmR-attR2 and CmR-pUC_ori fragments by PCR amplification from pRG2-ccdB and pGWC, with primers LoxGR2-XhF/ccdB-5R and Cm-PaF/pUC_ori-XhR, respectively. We purified the PCR fragments, digested them with *Xho*I and *Pac*I, and allowed them to ligate with each other, resulting in pGSR-P. We generated *SpR* fragment by PCR amplification from pPZP200 with primers Sp-F/R, we purified the PCR fragment, and ligated it with the *Xho*I-digested and T4 Pol-blunted pLM2BB-DOP, resulting in pSA. We generated *Apa*I and *Afl*II-flanked SpR-oriTF-R6K fragment by PCR amplification from pSA with primers Sp-ApR/R6K-AfR, we purified the PCR fragment, digested it with *Apa*I and *Afl*II, and inserted it into the *Apa*I and *Afl*II sites of pGSR-P, resulting in pGSR-P2. We inserted the Frt site generated by annealing two oligos oFrt-PXF/-PXR into the *Pac*I and *Xho*I sites of pGSP-P2, resulting in pGSR.

We transformed the pGSR into the DH10βF'DOT, we mixed the donor strain harboring pGSR and the heat-shocked recipient strain DH10B-Frt harboring pCP20, and allowed the conjugational transfer and Flp/Frt-mediated SSR reaction to occur, resulting in DH10B-RV.

To generate conjugationally transferrable plasmid harboring two sets of site-specific recombination proteins, we generated the oriTF element by PCR amplification from pMAGIC1with primers oriTF-SpF/BsR, we purified the PCR fragment, digested it with *Sph*I and *Bst*XI, and inserted it into the *Sph*I and *Bst*XI sites of pAH57-Cre, resulting in pAH57-Cre-TF. We transformed the pAH57-Cre-TF into the DH10βF'DOT. We mixed the DH10βF'DOT harboring pAH57-Cre-TF and strains harboring recipient vectors, allowed the conjugational transfer to occur, resulting in recipient strains, including AKG-pCB-LTR/RTL, and AGS-RV.

Creation of the engineered strain DH10B-SRP and recipient strains

We generated *N* fragment by PCR amplification from SW106[18] genomic DNA with primers N-PiScF0/F and N-ds-HiR, and we generated *xis-int* fragment by PCR amplification from pAH57-Cre[3] with primers Xis-up-EcF and Int-AiScR0/R. We purified the two PCR fragments, digested them with *Pac*I/*Hin*dIII, and *EcoR*I/*Asc*I, respectively. We ligated the *N* fragment with *Pac*I and *Hin*dIII-digested pLC-Amp, resulting in pLC-NA. We ligated the *xis-int* fragment with *Eco*RI and *Asc*I-digested pLC-NA, resulting in pLC-NAX. We digested pLC-NAX with *Hin*dIII and *Eco*RI, blunted the fragment with the T4 Pol, and allowed the fragment to self-ligate, resulting in pLC-NX. We generated the *N-xis-int* fragment from pLC-NX by digestion with I-SceI, and ligated with the I-SceI-digested pACBSR-A, resulting in pACBSR-NX.

We transformed pACBSR-NX into the *E. coli* strain SW106[18]. We conducted Gene Doctoring experiments according to the protocol[17] with modifications: we used one-plasmid strategy (pACBSR-NX) to replace two-plasmids strategy for Gene Doctoring experiments (pDOC and pACBSCE). We incubated the agar plates at 42°C to kill the background cells, resulting in the creation of engineered *E. coli* strain DH01B-SRP-attL, in which 5.9-kb fragment between *N* and *xis* genes was deleted by lambda RED-mediated homologous recombination.

We replaced *trfA-trfB* fragment between the *Hin*dIII and *Eco*RI sites of pDOC-YB-trfAB with an insert produced by annealing with two oligos oHSE-F/R, resulting in pDOC-YB2. We obtained the Frt-KmR-Frt fragment from pDOC-H by digestion with *Eco*RI and *Spe*I, and inserted the fragment into the *Eco*RI and *Spe*I sites of pDOC-YB2, resulting in pDOC-YB-K. We generated *int* fragment by PCR amplification from pAH57-Cre with primers int-PaF/HiR, and we generated rrnB-T1T2 by PCR amplification from pLC-ccdB with primers T1T2-HiF/SpR. We purified the two PCR fragments, digested them with *Pac*I/*Hin*dIII, and *Hin*dIII/*Spe*I, respectively. We ligated the two fragments with *Pac*I and *Spe*I-digested pDOC-YB-K to replace the *bioA*, resulting in pDOC-YI-K. We co-transformed pDOC-YI-K and pACBSCE into the *E. coli* strain DH01B-SRP-attL. We conducted Gene Doctoring experiments according to the protocol[17], resulting DH01B-SRP-FKF, in which Frt-KmR-Frt fragment replaced the attL site. We transformed pCP20 into DH10B-SRP-FKF, and allowed Flp/Frt-mediated SSR reaction to occur according to the protocol, resulting in DH10B-SRP. We transformed pCB-RTL/LTR into the DH10B-SRP, resulting in recipient strains, including KG-pCB-LTR/RTL.

Formulas for MISSA Reactions for Assembly of pABA61

(1) AKG-pCB-LTR + TEC-pLC2-Hyg → AKC-pABA61-R1
(2) AKC-pABA61-R1 + TEG-pRG2-NCED3 → AKG-pABA61-R2
(3) AKG-pABA61-R2 + TEC-pLC2-RCAR11 → AKC-pABA61-R3
(4) AKC-pABA61-R3 + TEG-pRG2-CBF3 → AKG-pABA61-R4
(5) AKG-pABA61-R4 + TEC-pLC2-GUS → AKC-pABA61-R5
(6) AKC-pABA61-R5 + TES-pSR1-TM1 → AKCS-pABA61 → AK-pABA61

See Tables 1 and 2 for nomenclature.

Formulas for MISSA Reactions for Assembly of pTEST51

(1) KG-pCB-LTR + TEC-pVLC-GUS → KC-pTEST51-R1
(2) KC-pTEST51-R1 + TEG-pVRG-gNHX8 → KG-pTEST51-R2
(3) KG-pTEST51-R2 + TEC-pVLC-Gly → KC-pTEST51-R3
(4) KC-pTEST51-R3 + TEG- pVRG-Hyg → KG-pTEST51-R4
(5) KG-pTEST51-R4 + TES-pSL2-iSc → KGS-pTEST51 → K-pTEST51

See Tables 1 and 2 for nomenclature.

Creation of the donor and recipient vectors for p2x3sgR

We disrupted *Bsa*I, *Ahd*I and two *BspH*I sites of ampicillin-resistance gene in pUC18, resulting in pUC18-mA. We amplified *ApR* gene with primer pair ApR-BBF/-BBR from pUC18-mA, digested the purified PCR products with *Bbs*I, and ligated with *Bsa*I-digested pCBC-U626p-gRS[14], resulting in pCBC-U6A. We amplified pCBC-U6A with three primers U6pt-NBF/-NBF0/-NBR, digested the purified PCR products, and self-ligated, resuling in pCBC-U6pt. We digested sgR@Sa, which was synthesized and cloned into pUC57 by GenScript (Nanjing), with *Bbs*I, and ligated the fragment with *Bsa*I-digested pCBC-U6pt, resulting in pCBC-U6sgR@Sa. We inserted the *Bsa*I fragment of *ApR* in pCBC-U6A into pCBC-sgR@Sa, resulting in pCBC-U6A@Sa. We first amplified two fragments: one with AsgR-F/26t29p-R from pCBC-U6A@Sa, and the other with 26t29p-F/29p-R from pCBC-DT1T2, and then we performed 2nd round PCR with AsgR-F/29p-R as primers and mixture of two PCR fragments as templates. We purified the PCR fragment and ligated with pCBC, resulting in pCBC-D12@Sa.

We replaced *Hin*dIII-*Nco*I fragment of pHEE401[19] with an insert formed by annealing oiCeuI-HNF/R, resulting in pNGG-P1. We amplified the U6-ApR-sgR cassette with three primers U6-BAF, U6-BEiCR0 and U6-BEiCR from pCBC-U6A, digested the purified PCR products with *Bsm*BI, and ligated with *Eco*RI-digested pNGG-P1,

resulting in pNGG. We amplified the U6-ApR-sgR@Sa cassette with three primers U6-BAF, U6-BEiCR0 and U6-BEiCR from pCBC-U6A@Sa, digested the purified PCR products with *Bsm*BI, and ligated with *Eco*RI-digested pNGG-P1, resulting in pNNGRRT-P1. We replaced *Xba*I-*Sac*I fragment of *zCas9* in pNNGRRT-P1 with *Xba*I-*Sac*I fragment of *zSaCas9*, which was codon-optimized with maize favored codons, synthesized, and cloned into pUC57 by GenScript (Nanjing), resulting in pNNGRRT.

We inserted the *Hin*dIII-*Eco*RI fragments of pNGG and pNNGRRT into the *Hin*dIII and *Eco*RI sites of pVLC-ccdB and pVRG-ccdB, resulting in pVLC-EPCU6A and pVRG-EPCU6A@Sa, respectively. We amplified germ-line-specific promoter *SPL*[20] with primers SPL-BNF/-BXR from *Arabidopsis* genomic DNA, digested the PCR products with *Bsa*I, and ligated with *Nco*I and *Xba*I-digested pVRG-EPCU6A@Sa to replace EC1.2en-EC1.1p, resulting in pVRG-GSCU6A@Sa. We amplified mCherry cassette with three primers mCherry-F0/-HMF/-EAR from pHDE 35S Cas9-mCherry[21], digested the purified PCR products, and ligated into *Hin*dIII and *Eco*RI sites of pVRG-ccdB, resuling in pVRG-mCherry. We amplified U6-ApR-sgRNA cassette with U6-ArF/-EcR from pCBC-U6A, digested the purified PCR products with *Avr*II and *Eco*RI, and inserted into the *Avr*II and *Eco*RI sites of pVRG-mCherry, resulting in pVRG-mCherryU6A.

We inserted an I-CeuI (homing endonuclease) site into the *Pac*I and *Hin*dIII sites of pVLC-ccdB, with an insert formed by annealing oPiCH-F/R, resulting in pVLC-ccdB-iC1. We inserted another I-CeuI site into the *Eco*RI and *Asc*I sites of pVLC-ccdB, with an insert formed by annealing oEiCA-F/R, resulting in pVLC-ccdB-iC. We amplified *Hyg* cassette with Hyg-HSF/-EcR from pHEE401E, digested with *Hin*dIII and *Eco*RI, and ligated with *Hin*dIII and *Eco*RI-digested pVLC-ccdB-iC, resulting in pVLC-Hyg-iC. We amplified LB region with LB-HiF/-SpR from pHEE401E, digested with *Hin*dIII and *Spe*I, and ligated with *Hin*dIII and *Spe*I-digested pVLC-Hyg-iC, resulting in pVLC-LBH. We amplified the U6-ApR-sgR cassette with primers U6-BAF/-BER from pCBC-U6A, digested the purified PCR products with *Bsm*BI, and ligated with *Eco*RI-digested pVLC-LBH, resulting in pVLC-LBHU6A.

We constructed pVLC-2sgR-1, pVRG-2sgR-2, and pVLC-2sgR-3 in the same manner as pHSE-2gR-CHLI[14] except that we used pVLC-EPCU6A, pVRG-EPCU6A@Sa, or pVLC-LBHU6A in place of pHSE401. In short, we obtained a single PCR fragment flanked by two sgRNA targets and two *Bsa*I sites incorporated by primers, we purified the PCR products, and digested them with *Bsa*I and ligated them with one of the *Bsa*I-

linearized donor vectors, resulting in the final donor vectors. To construct pVLC-2sgR-1, we used DT1-BsF/F0@T&C and DT2-R0/BsR@A1 as primers, and pCBC-DT1T2 as template DNA; To construct pVRG-2sgR-2, we used DT1-BsF/F0@Sa@T and DT2-R0/BsR@C as primers, and pCBC-D12@Sa as template DNA; To construct pVLC-2sgR-3, we used DT1-BsF/F0@Sa@A2 and DT2-R0/BsR@H1 as primers, and pCBC-D12 as template DNA.

We amplified *GmR* fragment with GmR-SaF/ApR2 from pUC-Em7G, digested the purified PCR products with *Sal*I and *Apa*I, and ligated with *Sal*I and *Apa*I-digested pR-ccdB[3], resulting in pCRG-ccdB. We amplified loxP-GmR-attR2 cassette with three primers RC-iCeuI-hStFR and loxGR2-iCeuF/R2 from pCRG-ccdB, purifed the PCR products, and ligated with *Pst*I and *Sac*II-digested and T4 DNA polymerase-blunted pHSEm[14], resulting in pMGW. We amplified RB-attR2-GmR-loxP-Kan fragment with primers RBGK-BsF/-BsR from pMGW, and cloned the purified PCR products into pCBC, resulting in pCBC-RBGK. We amplified oriV with primers oriV-BsF/-BbR from pXHL32, and oriT with primers oriT-BsF/-BbR from pL-oriT. We mixed the two PCR fragments, digested them with *Bbs*I, and ligated the purified products with pCBC, resulting in pCBC-oriVT. We amplified pVS1 fragment with primers VS1-BsF22/-BsR22 from pHSEm[14], and cloned the purified PCR products into pCBC, resulting in pCBC-VS1. We performed Golden Gate assembly with pCBC-RBGK, pCBC-oriVT, and pCBC-VS1 digested with *Bsa*I, resulting in pKTV. We inserted the linearized pKTV by *Nru*I digestion into two *Nru*I sites of pBeloBAC11, resulting in pCB-RTL2. We introduced the recipient vector pCB-RTL2 into DH10B-SRP and the three final donor vectors (pVLC-2sgR-1, pVRG-2sgR-2, and pVLC-2sgR-3) into P254D-TE, and performed 3 rounds of MISSA reactions, resulting in p2x3sgR.

Generation of CRISPR/Cas9 transgenic *Arabidopsis* and analysis of mutations
We transformed the p2x3sgR into *Agrobacterium* strain GV3101. We transformed *Arabidopsis* Col-0 wild-type plants via the floral dip method[22]. We screened the collected seeds from the T0 plants on MS plates containing 25 mg/L hygromycin, and transplanted the resistant seedlings (T1) to soil. We extracted genomic DNA from T1 or T2 transgenic plants grown in soil. To analyze mutations of *TRY*, *CPC* and *ABI2*, we amplified fragments spanning the two target sites of *TRY*, *CPC* or *ABI2* by PCR using gene-specific primers TRY-5U-F/-3U-R, CPC-5U-F/-3Uds-R, or ABI2-IDF0/-IDR0, respectively. To analyze mutations of *ABI1* and *HAB1*, we amplified fragments spanning the target sites of *ABI1* or HAB1 by PCR using gene-specific primers ABI1-IDF0/-IDR0 or HAB1-IDF/-IDR, respectively. To analyze mutations of *TRY*, *CPC*, *ABI2*, and *HAB1*, we submitted purified PCR products for direct sequencing with the

same primers as those for PCR. We analyzed mutations of *ABI1* by *Nco*I digestion analysis of the PCR fragment.

To analyze possible mutations of potential off-target sites of *AT5G02760*, *AT2G25070*, and *AT3G17090* of the sgRNA targeting *ABI1*, we amplified fragments surrounding the off-target sites by PCR using gene-specific primers AT5G02760-F/R, AT2G25070-F/R, or AT3G17090-F/R, respectively. We analyzed off-target mutations of *AT5G02760* and *AT2G25070* by *Nco*I digestion analysis of the PCR fragments. To analyze mutations of *AT3G17090*, we submitted purified PCR products for direct sequencing with the same primers as those for PCR.

References

1. Li, M. Z. & Elledge, S. J. MAGIC, an in vivo genetic method for the rapid construction of recombinant DNA molecules. *Nat. Genet.* **37**, 311-319, doi:10.1038/ng1505 (2005).
2. Chen, Q. J., Zhou, H. M., Chen, J. & Wang, X. C. Using a modified TA cloning method to create entry clones. *Anal. Biochem.* **358**, 120-125, doi:10.1016/j.ab.2006.08.015 (2006).
3. Chen, Q. J. *et al.* MISSA is a highly efficient in vivo DNA assembly method for plant multiple-gene transformation. *Plant Physiol.* **153**, 41-51, doi:10.1104/pp.109.152249 (2010).
4. Brand, L. *et al.* A versatile and reliable two-component system for tissue-specific gene induction in Arabidopsis. *Plant Physiol.* **141**, 1194-1204, doi:10.1104/pp.106.081299 (2006).
5. Curtis, M. D. & Grossniklaus, U. A gateway cloning vector set for high-throughput functional analysis of genes in planta. *Plant Physiol.* **133**, 462-469, doi:10.1104/pp.103.027979 (2003).
6. Yang, Y., Costa, A., Leonhardt, N., Siegel, R. S. & Schroeder, J. I. Isolation of a strong Arabidopsis guard cell promoter and its potential as a research tool. *Plant Methods* **4**, 6, doi:10.1186/1746-4811-4-6 (2008).
7. Ma, Y. *et al.* Regulators of PP2C phosphatase activity function as abscisic acid sensors. *Science* **324**, 1064-1068, doi:10.1126/science.1172408 (2009).
8. Park, S. Y. *et al.* Abscisic acid inhibits type 2C protein phosphatases via the PYR/PYL family of START proteins. *Science* **324**, 1068-1071, doi:10.1126/science.1173041 (2009).
9. Kasuga, M., Liu, Q., Miura, S., Yamaguchi-Shinozaki, K. & Shinozaki, K. Improving plant drought, salt, and freezing tolerance by gene transfer of a single stress-inducible transcription factor. *Nat. Biotechnol.* **17**, 287-291, doi:10.1038/7036 (1999).
10. Gilmour, S. J., Sebolt, A. M., Salazar, M. P., Everard, J. D. & Thomashow, M. F. Overexpression of the Arabidopsis CBF3 transcriptional activator mimics

multiple biochemical changes associated with cold acclimation. *Plant Physiol.* **124**, 1854-1865 (2000).
11. Iuchi, S. *et al.* Regulation of drought tolerance by gene manipulation of 9-cis-epoxycarotenoid dioxygenase, a key enzyme in abscisic acid biosynthesis in Arabidopsis. *Plant J.* **27**, 325-333 (2001).
12. An, R. *et al.* AtNHX8, a member of the monovalent cation: proton antiporter-1 family in Arabidopsis thaliana, encodes a putative Li/H antiporter. *Plant J.* **49**, 718-728, doi:10.1111/j.1365-313X.2006.02990.x (2007).
13. Hajdukiewicz, P., Svab, Z. & Maliga, P. The small, versatile pPZP family of Agrobacterium binary vectors for plant transformation. *Plant Mol. Biol.* **25**, 989-994 (1994).
14. Xing, H. L. *et al.* A CRISPR/Cas9 toolkit for multiplex genome editing in plants. *BMC Plant Biol.* **14**, 327, doi:10.1186/s12870-014-0327-y (2014).
15. Haldimann, A. & Wanner, B. L. Conditional-replication, integration, excision, and retrieval plasmid-host systems for gene structure-function studies of bacteria. *J. Bacteriol.* **183**, 6384-6393, doi:10.1128/JB.183.21.6384-6393.2001 (2001).
16. Hamilton, C. M. A binary-BAC system for plant transformation with high-molecular-weight DNA. *Gene* **200**, 107-116 (1997).
17. Lee, D. J. *et al.* Gene doctoring: a method for recombineering in laboratory and pathogenic Escherichia coli strains. *BMC Microbiol.* **9**, 252, doi:10.1186/1471-2180-9-252 (2009).
18. Warming, S., Costantino, N., Court, D. L., Jenkins, N. A. & Copeland, N. G. Simple and highly efficient BAC recombineering using galK selection. *Nucleic Acids Res.* **33**, e36, doi:10.1093/nar/gni035 (2005).
19. Wang, Z. P. *et al.* Egg cell-specific promoter-controlled CRISPR/Cas9 efficiently generates homozygous mutants for multiple target genes in Arabidopsis in a single generation. *Genome Biol.* **16**, 144, doi:10.1186/s13059-015-0715-0 (2015).
20. Mao, Y. *et al.* Development of germ-line-specific CRISPR-Cas9 systems to improve the production of heritable gene modifications in Arabidopsis. *Plant Biotechnol. J.* **14**, 519-532, doi:10.1111/pbi.12468 (2016).
21. Gao, X., Chen, J., Dai, X., Zhang, D. & Zhao, Y. An Effective Strategy for Reliably Isolating Heritable and Cas9-Free Arabidopsis Mutants Generated by CRISPR/Cas9-Mediated Genome Editing. *Plant Physiol.* **171**, 1794-1800, doi:10.1104/pp.16.00663 (2016).
22. Clough, S. J. & Bent, A. F. Floral dip: a simplified method for Agrobacterium-mediated transformation of Arabidopsis thaliana. *Plant J.* **16**, 735-743 (1998).

Supplementary File S1: Please See the ZIP file**.**
